# Crystal structures of bis- and hexakis[(6,6′-di­hydroxy­bipyridine)copper(II)] nitrate coordination complexes

**DOI:** 10.1107/S205698901502037X

**Published:** 2015-11-04

**Authors:** Deidra L. Gerlach, Ismael Nieto, Corey J. Herbst-Gervasoni, Gregory M. Ferrence, Matthias Zeller, Elizabeth T. Papish

**Affiliations:** a Department of Chemistry, University of Alabama, 250 Hackberry Lane, Tuscaloosa, AL 35487-0336, USA; b Department of Chemistry, Drexel University, 3141 Chestnut St., Philadelphia, PA 19104, USA; c Department of Chemistry, Illinois State University, Campus Box 4160, Normal, IL 61790-4160, USA; d Department of Chemistry, Youngstown State University, One University Plaza, Youngstown, OH 44555, USA

**Keywords:** crystal structure, copper, di­hydroxy­pyridine, potential water oxidation catalysts

## Abstract

Two multinuclear complexes synthesized from Cu(NO_3_)_2_ and 6,6′-di­hydroxy­bipyridine (dhbp) exhibit bridging nitrate and hydroxide ligands. The dinuclear complex (6,6′-di­hydroxy­bipyridine-2κ^2^ *N*,*N*′)[μ-6-(6-hy­droxy­pyridin-2-yl)pyridin-2-olato-1:2κ^3^ *N*,*N*′:*O* ^2^](μ-hydroxido-1:2κ^2^ *O*:*O*′)(μ-nitrato-1:2κ^2^ *O*:*O*′)(nitrato-1κ*O*)dicopper(II), [Cu_2_(C_10_H_7_N_2_O_2_)(OH)(NO_3_)_2_(C_10_H_8_N_2_O_2_)] or [Cu(6-OH-6′-O-bpy)(NO_3_)(μ-OH)(μ-NO_3_)Cu(6,6′-dhbp)], (I), with a 2:1 ratio of nitrate to hydroxide anions and one partially deprotonated dhbp ligand, forms from a water–ethanol mixture at neutral pH. The hexa­nuclear complex bis­(μ_3_-bi­pyridine-2,2′-diolato-κ^3^ *O*:*N*,*N*′:*O*′)tetra­kis­(6,6′-di­hydroxy­bipyridine-κ^2^ *N*,*N*′)tetra­kis­(μ-hydroxido-κ^2^ *O*:*O*′)bis­(methanol-κ*O*)tetra­kis­(μ-nitrato-κ^2^ *O*:*O*′)hexa­copper(II), [Cu_6_(C_10_H_6_N_2_O_2_)_2_(CH_4_O)_2_(OH)_4_(NO_3_)_4_(C_10_H_8_N_2_O_2_)_4_] or [Cu(6,6′-dhbp)(μ-NO_3_)_2_(μ-OH)Cu(6,6′-O-bpy)(μ-OH)Cu(6,6′dhbp)(CH_3_OH)]_2_, (II), with a 1:1 NO_3_–OH ratio and two fully protonated and fully deprotonated dhbp ligands, was obtained by methanol recrystallization of material obtained at pH 3. Complex (II) lies across an inversion center. Complexes (I) and (II) both display intra­molecular O—H⋯O hydrogen bonding. Inter­molecular O—H⋯O hydrogen bonding links symmetry-related mol­ecules forming chains along [100] for complex (I) with π-stacking along [010] and [001]. Complex (II) forms inter­molecular O—H⋯O hydrogen-bonded chains along [010] with π-stacking along [100] and [001].

## Abstract 2

Two copper(II) complexes, a dinuclear and a hexa­nuclear complex with bridging hydroxyl and nitrate ligands, were obtained from reaction of copper nitrate with di­hydroxy­bipyridine at neutral and slightly acidic pH. Formation of multi-nuclear complexes contrasts with the equivalent sulfate compounds which formed discrete mononuclear complexes. The complexes feature intra­molecular and inter­molecular hydrogen bonding.

## Chemical context   

Catalytic processes in nature are often facilitated by enzymes that feature transition metals in their active sites. Many of these reactions would be of tremendous inter­est could they be copied using simpler and technologically feasible conditions. One such process is water oxidation as observed in photosynthesis, and the use of transition metal complexes to mimic the reactivity of photosystem II have captured the attention of an increasing number of research groups over the last few years (Kikuchi & Tanaka, 2014 ▸; Singh & Spiccia, 2013 ▸). One complex that especially caught our inter­est was [Cp*Ir(bpy)Cl]^+^ (Blakemore *et al.*, 2010 ▸) which features a bi­pyridine (bpy) type ligand. In our research into catalytic water oxidation, we are trying to enhance proton-coupled electron transfer (PCET) in metal-complex catalysts by incorporating hydrogen-bond donors and acceptors in near proximity to the potentially catalytic metal atoms to mimic the active center of a protein–metal complex. When applying this principal to the Blakemore-type [Cp*Ir(bpy)Cl]^+^ complex by swapping normal bi­pyridine for di­hydroxy­bipyridine (6,6′-dhbp), we were indeed able to increase the catalytic turnover rate and control water oxidation rates by adjusting pH levels (DePasquale *et al.*, 2013 ▸). The ligand 6,6′-dhbp has also been used in combination with ruthenium terpyridine (tpy) fragments to yield the complex [(tpy)Ru(6,6′-dhbp)(H_2_O)] (Marelius *et al.*, 2014 ▸).
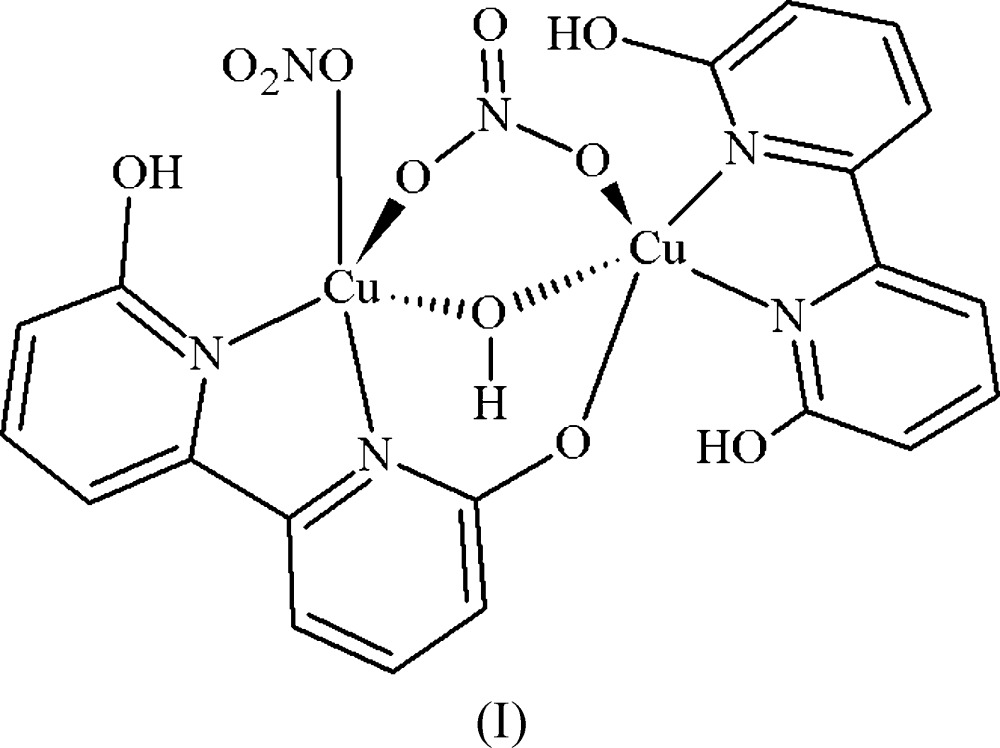
 
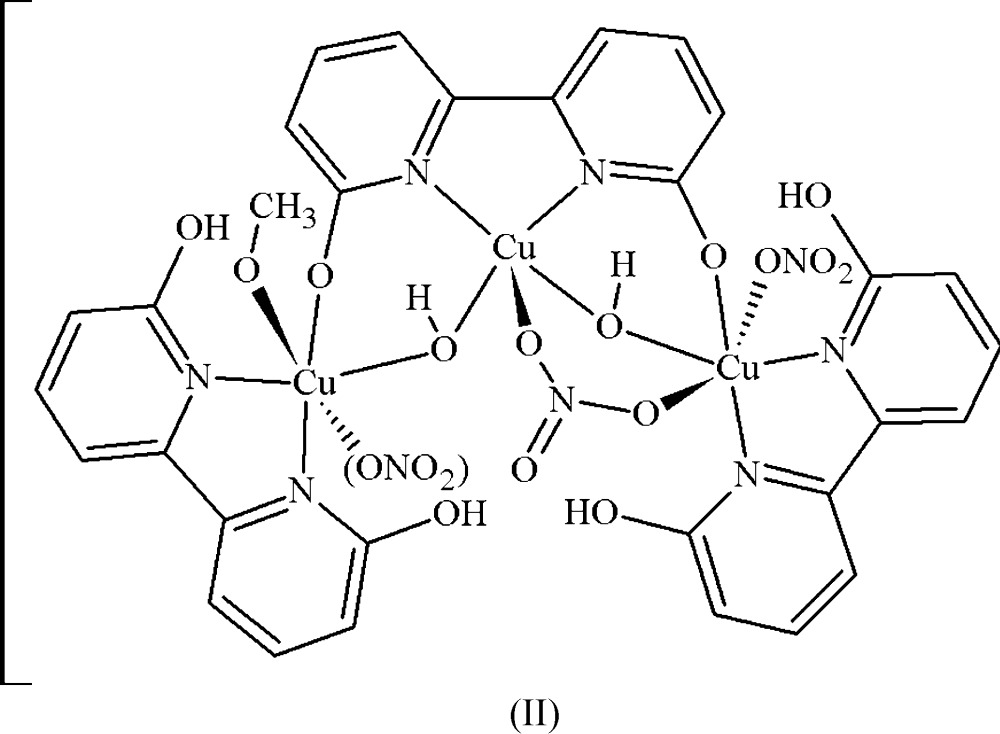


Our focus has most recently shifted to the investigation of copper(II) bi­pyridine complexes analogous to [(bpy)Cu(OH)_2_]_2_^2+^ (Barnett *et al.*, 2012 ▸). We isolated discrete mono-copper complexes from copper(II) sulfate with a selection of modified bi­pyridine ligands and investigated the compounds spectroscopically, crystallographically and for their catalytic water oxidation capacity (Gerlach *et al.*, 2014 ▸). When swapping sulfate for nitrate as the counter-ion we found that the resulting complexes are no longer mononuclear. Instead, larger aggregates with two or six copper(II) atoms formed that feature coordinating nitrate as well as hydroxyl ligands. As a result of their aggregation and the varied coordination environment of their copper atoms, these complexes are not ideally suited for homogenous water oxidation catalysis. Instead they feature quite intriguing and fascinating solid structures which we would like to describe and present.

## Structural commentary   

**Figure 1:**
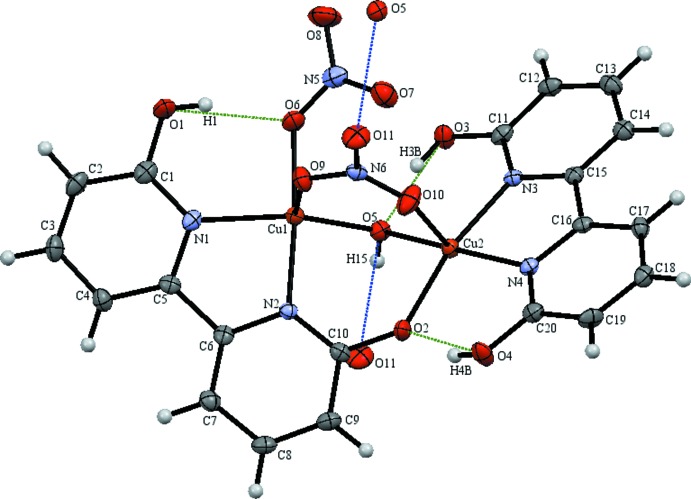
The numbering scheme of complex (I), with donor–acceptor distances of intra­molecular hydrogen bonds colored green and of inter­molecular hydrogen bonds colored blue, represented with displacement ellipsoids at the 50% probability level. Additional symmetry-related atoms O5 and O11 were generated by translation along the *a* axis.

The dinuclear copper(II) dhbp complex (I) contains nitrate as a co-ligand with both a fully protonated and a mono-deprotonated dhbp ligand (see Fig. 1 ▸). Two unique 6,6′-dhbp binding modes were observed for this copper(II) nitrate complex illustrating the structural flexibility of this ligand. The [6,6′-(OH)_2_bpy] ligand exhibits the typically observed bi­pyridine (*N*,*N*) coordination mode through N3 and N4 binding to Cu2. A new coordination mode is observed for the mono-deprotonated (6-O-6′-OH-bpy) ligand in which it bridges two metals, through (*N*,*N*) coordination of N1,N2 to Cu1 and through bridging to Cu2 *via* the pyridino­late oxygen O2 [1.946 (3) Å]. The C—O bond lengths (Å) for the dhbp ligands are 1.335 (5) (C1—O1), 1.322 (5) (C11—O3), and 1.316 (4) (C20—O4) for the protonated hydroxyl groups and slightly shorter at 1.310 (4) (C10—O2) for the pyridino­late, reflecting double-bond character. Both copper atoms have a distorted square-pyramidal geometry with τ = 0.394 at Cu1 and τ = 0.119 at Cu2 (Addison *et al.*, 1984 ▸). This structure has a close Cu⋯Cu distance of 3.158 (9) Å. Complex (I) features three strong intra­molecular hydrogen bonds (Jeffrey, 2003 ▸) with O⋯O distances (Å) as follows: 2.583 (4) for O1 to O6 (dhbp to nitrate) with bond angle O—H⋯O of 163 (5)°, 2.528 (4) for O4 to O2 (dhbp to deprotonated dhbp) with bond angle of 161 (5)°, and 2.510 (4) for O3 to O5 (dhbp to hydrox­yl) with bond angle of 164 (5)°. One inter­molecular hydrogen bond tethers one dimer of complex (I) to the next in a head-to-tail fashion *via* a 2.802 (4) Å hydrogen bond from O5 to O11 (hydroxyl to nitrate) with a bond angle of 164 (4)°. Numerical details of the hydrogen bonds are given in Table 1 ▸.

**Figure 2:**
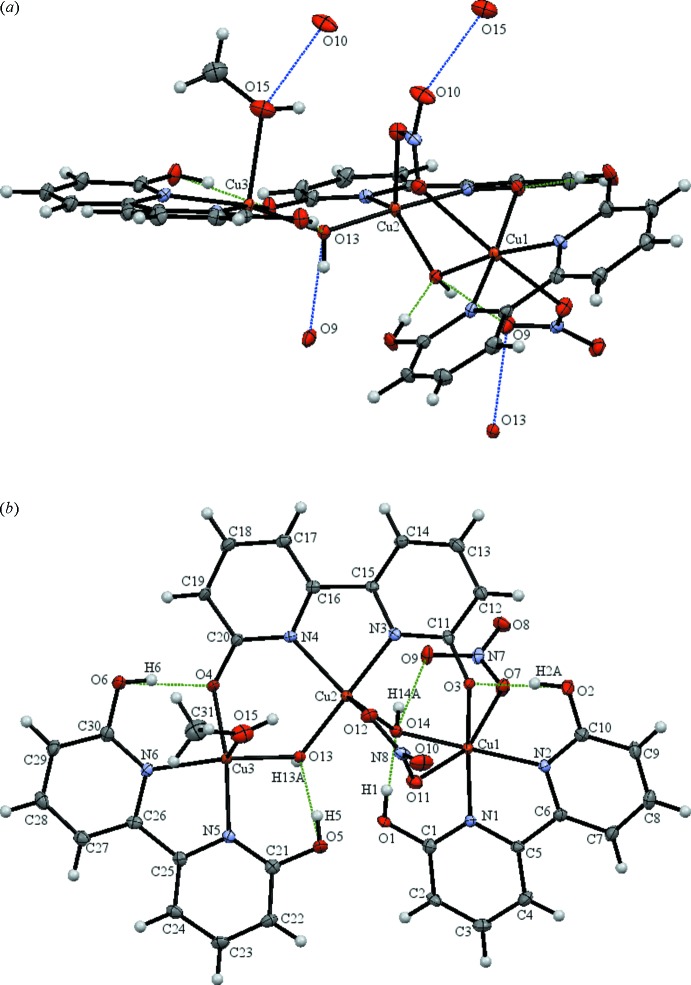
(*a*) The asymmetric unit of complex (II) represented with displacement ellipsoids at the 50% probability level. H atoms not involved in hydrogen bonding are omitted for clarity. The donor–acceptor distances are shown as green for intra­molecular hydrogen bonds and shown as blue for inter­molecular hydrogen bonds (O10–O15) and inter-asymmetric unit hydrogen bonds (O9 –O13). Additional symmetry-related atoms O10 and O15 were generated by the symmetry operator 1 − *x*, −*y*, 1 − *z*, and O9 and O13 were generated *via* the inversion center 1 − *x*, 1 − *y*, 1 − *z* at the center of the hexa­nuclear complex. (*b*) The asymmetric unit of complex (II) oriented to show donor–acceptor distances of intra­molecular hydrogen bonds, in green, of the protonated dhbp ligands.

**Figure 3:**
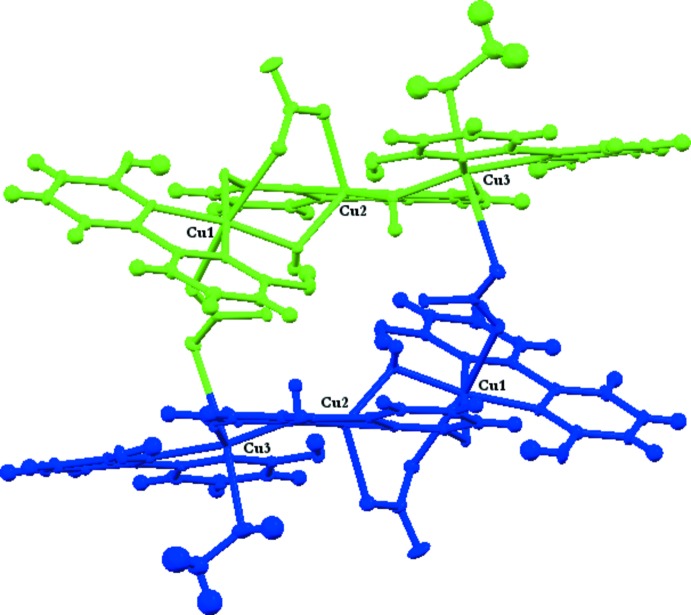
The full hexanuclear complex (II) represented with displacement ellipsoids at the 50% probability level and H atoms not involved in hydrogen bonding are omitted for clarity. The two symmetry-related units of the hexa­mer are shown in blue and green to better visualize the relation of the asymmetric unit through the inversion center.

**Figure 4:**
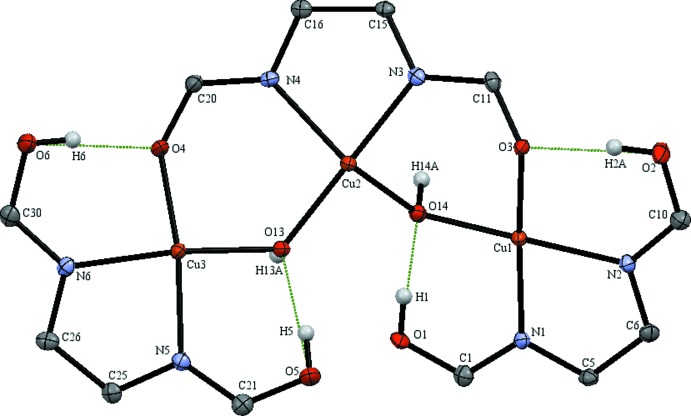
The three unique copper atoms of complex (II) are displayed with the coordination relevant atoms of the bound dhbp ligands and bridging hydroxides with displacement ellipsoids at the 50% probability level and the donor–acceptor distances of intra­molecular hydrogen bonds of the protonated dhbps represented in green.

The hexa­nuclear copper(II) dhbp complex (II) is comprised of a dimer of the asymmetric portion of the molecule which contains three symmetry-unique copper atoms, two fully protonated and one fully deprotonated dhbp, two bridging hydroxide and two nitrate ligands (see Fig. 2 ▸). This asymmetric trinuclear unit is related through an inversion center to the full hexa­nuclear complex (see Fig. 3 ▸). Two copper atoms, Cu1 and Cu3, are hexa-coordinate with a distorted octa­hedral geometry whereas Cu2 is penta-coordinate with a distorted trigonal–pyramidal geometry with τ = 0.746 (Addison *et al.*, 1984 ▸). Similar to the dinuclear complex, each copper atom is coordinated by one hy­droxy­bipyridine ligand with one bridging hydroxyl ligand between Cu1 to Cu2 and Cu2 to Cu3. The di­hydroxy­bipyridine ligand bound to Cu2 (dhbp2) is doubly deprotonated with each deprotonated oxygen bound to the flanking Cu1 and Cu3 metal sites, O3 and O4, respectively. The remaining coordination sphere of Cu1 entails one dhbp (*N*,*N* bound), one bridging hydroxide to Cu2 (O14), one bridging nitrate to Cu2 (O11), and one bridging nitrate (O7) which tethers the two asymmetric units. The coordination of Cu2 entails one deprotonated dhbp (*N*,*N* bound), one bridg­ing nitrate to Cu1 (O12), and two bridging hydroxides to Cu1 and Cu3 (O14 and O13, respectively). The remaining coord­ination sphere of Cu3 entails one dhbp (*N*,*N* bound), one methanol (O15), one bridging hydroxide (O13), and one bridging nitrate (O8) which tethers the two asymmetric units. Each deprotonated oxygen of dhbp acts as an acceptor for intra­molecular hydrogen bonds from the two protonated dhbp ligands, O2 to O3 at 2.499 (3) Å (O—H⋯O bond angle of 172°), and O6 to O4 at 2.495 (3) Å (O—H⋯O bond angle of 168°) shown in Fig. 4 ▸. The remaining hydroxyl groups of the protonated dhbps form strong hydrogen bonds to the bridging hydroxides where O1 donates to O14 [2.448 (3) Å, O—H⋯O bond angle of 175°] of the hydroxide bridging Cu1 and Cu2, and O5 donates to O13 [2.536 (3) Å, O—H⋯O bond angle of 170°] of the hydroxide bridging Cu2 and Cu3 (Table 2 ▸). Inter­estingly, both Cu—Cu bridging hydroxides form an inter­mediate strength intra­molecular hydrogen bond to the bridging nitrate linking the two asymmetric units of the hexa­mer, with O⋯O distances (Å) of O13 and O14 to O9 being 2.763 (3) [O—H⋯O angle of 158 (4)°] and 2.738 (3) [O—H⋯O angle of 163 (4)°], respectively. C—O bond lengths of the protonated and deprotonated dhbp ligands of the hexa and dinuclear complexes are similar; C⋯O distances (Å) are 1.302 (4) and 1.312 (4) for the copper coordinating oxygen atoms, and 1.324 (4), 1.304 (4), 1.330 (4), and 1.321 (4) for the hydroxyl O atoms, with the longer of the four values belonging to the hydroxyl groups hydrogen-bound to the neighboring deprotonated dhbp ligand, and the shorter two being associated with those hydrogen-bound to the bridging hydroxyl groups. These reduced lengths reflect increased C—O double-bond character upon deprotonation. One inter­molecular hydrogen bond of inter­mediate strength connects the bound methanol to the non-coordinating oxygen of the Cu1–Cu2 bridging nitrate of another mol­ecule, O15 to O10 at 2.790 (4) Å [O—H⋯O bond angle of 107 (4)°].

**Figure 5:**
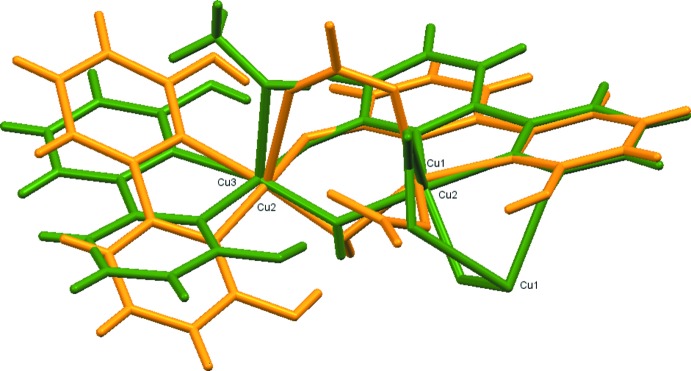
Complex (I) in yellow is overlaid on the Cu2—Cu3 dimer portion of the asymmetric unit of complex (II) in green.

Comparison of complex (I) to the asymmetric component of complex (II) indicates some structural similarities, Fig. 5 ▸. The overall structure of complex (I) can be reasonably well overlaid with the dinuclear component of (II) including Cu2 and Cu3, with the main differences resulting from one nitrate ligand that is bridging between the two copper ions in complex (I) being rotated so that in complex (II) it instead bridges one of these copper ions to the third that has no counterpart in complex (I). The two copper ions that are bridged by a nitrate ion in complex (I) are thus not bridged in complex (II) (featuring a methanol mol­ecule and a nitrate bridging to the third copper instead), leading to a larger distance between the copper ions in complex (II) and a different tilt angle of the fully protonated dhbp ligand (left dhbp in Fig. 5 ▸).

The bridging oxygen species for both complexes (I) and (II) are correctly assigned as hydroxides to balance the overall neutral charge of the complex. Complex (I) with two Cu^II^ ions is charge balanced with one terminal and one bridging nitrate each with a single negative charge, one deprotonated hydroxyl group of dhbp, and one bridging hydroxide. The bond lengths to the bridging hydroxide from Cu1 to O5 is 1.964 (3) Å and Cu2 to O5 is 1.939 (3) Å where the proton of O5 hydrogen bonds to one acceptor. A comparable bond length is 1.946 (3) Å from Cu2 to O2 of the deprotonated dhbp ligand. The remaining oxygen atoms of the dhbp ligands are each protonated and engaged in hydrogen bonding as described above. The asymmetric unit of complex (II) balances similarly with three Cu (II) ions against one bridging nitrate, one nitrate bridging the two asymmetric units, two deprotonated dhbp hydroxyl groups, and two bridging hydroxides. The bond lengths to the bridging hydroxide, Cu2 and Cu3 to O13: 1.933 (2) and 1.951 (2) Å, respectively, are comparable to those observed in complex (I) where each of these hydroxides have one hydrogen bond. Alternatively, the bond lengths to the bridging hydroxide are longer where Cu1 and Cu2 to O14 are 1.970 (2) and 2.062 (3) Å, respectively, likely due to the two hydrogen-bonding inter­actions described above weakening the orbital overlap with the copper and lengthening these bonds. The two copper–hydroxyl dhbp bonds in complex (II) are comparable to this bond in complex (I) at 1.966 (3) Å from Cu1 to O3 of dhbp and 1.960 (2) Å from Cu3 to O4 of dhbp.

## Supra­molecular features   

**Figure 6:**
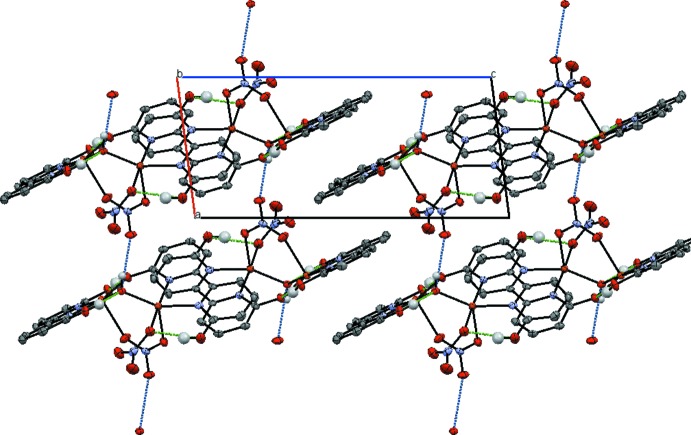
The packing arrangement of complex (I) propagates along [100] *via* inter­molecular hydrogen bonding (blue) and in the *bc* plane by π-stacking of the dhbp pyridyl rings.

Some inter­molecular hydrogen-bonding inter­actions in both complexes have already been discussed, *vide supra*. The dinuclear complex (I) also features inter­molecular parallel offset π-stacking of both dhbp ligands. The dhbp ligand coordinating to Cu1 is π-stacked with its symmetry counterpart alternating across two inversion centers, one for each ring of this dhbp ligand. These alternating π–π inter­actions form chains in the [010] direction. The pyridine ring containing N2 inter­acts with the symmetry-equivalent ring of a neighboring mol­ecule across the symmetry operation 1 − *x*, −*y*, −*z* at a distance of 3.894 (3) Å between the centroids of the rings. The pyridine ring containing N1 inter­acts with its symmetry-equivalent ring across the symmetry operation 1 − *x*, 1 − *y*, −*z* with a centroid-to-centroid distance of 3.969 (3) Å. The dhbp ligand coordinating to Cu2 also shows π-stacking *via* two alternating inversion-symmetry operations, forming chains along [100]. The pyridine rings containing N3 and N4 inter­cross by the symmetry operation 1 − *x*, −*y*, 1 − *z* where the centroid of the ring defined by N3 is at a distance of 3.604 (2) Å from the centroid of the ring defined as N4 on side of the dhbp plane with the bridging hydroxide. These rings also π-stack on the opposite face of the plane at a distance of 3.768 (2) Å from the centroid of the ring defined by N3 to N4 through the symmetry operation −*x*, −*y*, 1 − *z*. Inter­molecular hydrogen bonding from the bridging hydroxide ligand to the terminal oxygen of the bridging nitrate ligand inter­links neighboring mol­ecules primarily along [100]. See Fig. 6 ▸ for extended inter­molecular inter­actions of complex (I).

**Figure 7:**
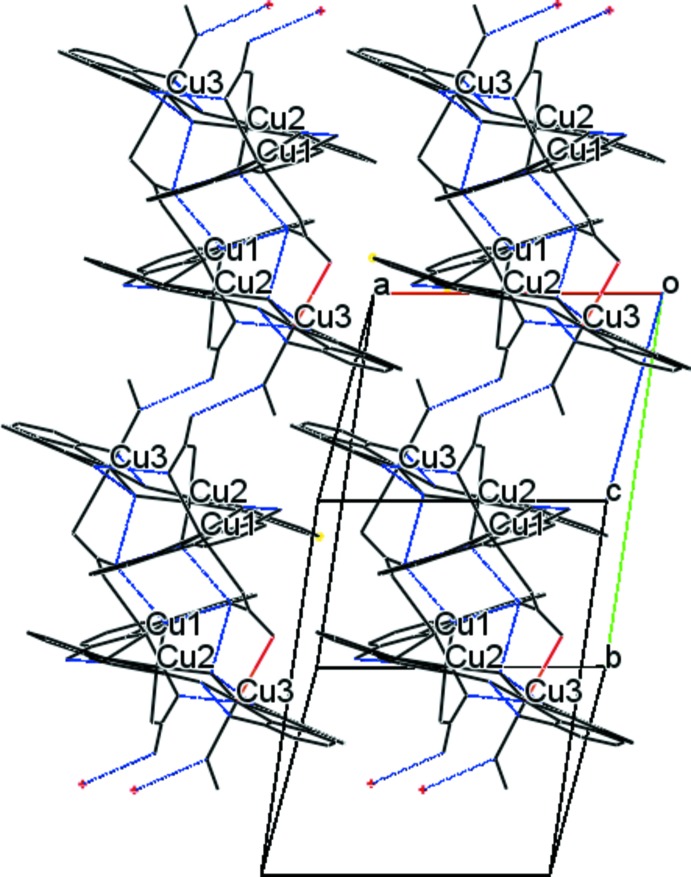
The packing arrangement of complex (II) propagates along [010] *via* inter­molecular hydrogen bonding (blue) and in the *ac* plane by π-stacking of the dhbp pyridyl rings.

The hexa­nuclear complex (II) progresses along [010] through two symmetry-related hydrogen bonds between O15 of the bound methanol mol­ecule of Cu3 to O10 of the Cu1–Cu2 bridging nitrate (Fig. 7 ▸). The dhbp ligands are primarily within the *ac* plane and exhibit π-stacking but in a less regular fashion than for complex (I), primarily in the [010] direction without forming chains. Off-set π-stacking of the dhbp ligand bound to Cu1 are related through the symmetry operation 1 − *x*, −*y*, −*z* with a centroid-to-centroid distance of 3.784 (2) Å of the pyridine rings containing N1 to the ring containing N2 and *vice versa*. A single ring of each dhbp ligand bound to Cu2 and Cu3 π-stack *via* translation at a distance of 3.551 (2) Å between the centroids of the pyridine rings defined by N3 and N5, respectively. Additionally, the Cu3 dhbp ligand π-stacks *via* the symmetry-equivalent ring defined by N5 of a neighboring mol­ecule across the symmetry operation −*x*, −*y*, 1 − *z* at a centroid-to-centroid distance of 3.887 (2) Å. Close proximity occurs in plane between the pyridine ring containing N4 of the dhbp ligand bound to Cu2 at a distance of 3.818 (2) Å between C18 to C19 and *vice versa* across the symmetry operation 1 − *x*, 1 − *y*, −*z*.

## Database survey   

Although many structures have been reported featuring a hydroxide anion bridging two copper(II) ions each bound by 2,2′-bi­pyridine, no analogous structure has been reported with a 6,6′-dihy­droxy-2,2′-bi­pyridine ligand. A search of the Cambridge Structural Database (Version 5.36, May 2015; Groom & Allen, 2014 ▸) for the substructure of copper ligated by 6-hy­droxy-2,2′-bi­pyridine, where the hydroxyl group (–OH) further ligates to a second copper atom, resulted in several structures. These primarily planar structures are reported either with co-crystallized metal-containing counter-ions: CSD refcode QEXHUX (Guo *et al.*, 2007 ▸), VIHZIX (Zhong, Li *et al.*, 2013 ▸), WUJGUE (Wang *et al.*, 2009 ▸), XIQGAH (Zhong, Feng *et al.*, 2013 ▸); or with a bridging mol­ecule linking two of these planar copper dimers: IYOWOI (He & Lu, 2004 ▸), MISPUZ (Zhang, Tong & Chen, 2002 ▸), REMMAY (Luo *et al.*, 2006 ▸), SESDAW (Sun *et al.*, 2006 ▸), XOVTEH (Zhang, Tong, Gong *et al.*, 2002 ▸).

The most relevant structure reported in the database contains a dinuclear copper 6-hy­droxy­bipyridine complex with a nitrate ligand bridging the copper ions, IBOXAZ (Zhang *et al.*, 2004 ▸). No examples of hy­droxy­bipyridine-ligated copper compounds with oxide or hydroxide bridges have been reported.

## Synthesis and crystallization   

***The neutral copper dinuclear complex* (I)** Copper(II) nitrate trihydrate (128 mg, 0.530 mmol) and 6,6′-dhbp (100 mg, 0.531 mmol) were combined in 50/50 ethanol and water solvent (10 mL) and stirred two days. Green plate crystals were grown from an ethanol solution in a freezer. This complex was analyzed exclusively by X-ray diffraction.

***The neutral copper hexanuclear complex* (II)** Copper(II) nitrate hemi­penta­hydrate (124 mg, 0.533 mmol) and 6,6′-dhbp (100 mg, 0.531 mmol) were combined in 10 mL of 0.1 M NaOAc adjusted to pH 3 by acetic acid. The mixture was stirred for three days at room temperature. The resulting solution was dried under high vacuum and recrystallized twice from methanol to afford green prismatic crystals. This complex was analyzed exclusively by x-ray diffraction.

## Refinement   

Crystal data, data collection, and structure refinement details are summarized in Table 3 ▸.

The crystal under investigation for complex (II) was found to be split with two major domains not related by any obvious twin operation. The orientation matrices for the two components were identified using the program *Cell Now* (Sheldrick, 2008 ▸), with the two components being related by a 2.9° rotation about either the reciprocal axis 1.000 − 0.363 − 0.339 or the real axis 1.000 − 0.178 − 0.265. The two components were integrated using *SAINT* (Bruker, 2012 ▸), resulting in the following statistics: 17535 data (5769 unique) involve domain 1 only, mean *I*/σ 8.6, 17271 data (5689 unique) involve domain 2 only, mean *I*/σ 8.2, 34813 data (9811 unique) involve 2 domains, mean I/sigma 9.5, 11 data (11 unique) involve 3 domains, mean *I*/σ 8.7 and 4 data (2 unique) involve 4 domains, mean *I*/σ 57.6 The exact correlation matrix as identified by the integration program was found to be 1.00336 0.02923 −0.02720, −0.01894 1.02272 −0.04903, 0.02520 0.05747 0.97055. The data were corrected for absorption using *TWINABS* (Sheldrick, 2009 ▸), and the structure was solved using direct methods with only the non-overlapping reflections of component 1. The structure was refined using the HKLF5 routine with all reflections of component 1 (including the overlapping ones), resulting in a BASF value of 0.486 (1). The *R*_int_ value given is for all reflections and is based on agreement between observed single and composite intensities and those calculated from refined unique intensities and twin fractions (*TWINABS*; Sheldrick, 2009 ▸).

C- and O-bound H atoms were placed in calculated positions and allowed to ride on their carrier atoms: aromatic C—H_arom_ = 0.95 Å with *U*_iso_(H) = 1.2*U*_eq_(C), C—H_meth­yl_ = 0.98 Å with *U*_iso_(H) = 1.5*U*_eq_(C). O—H were refined for complex (I) and for hydroxide and methanol H atoms of complex (II), with O—H distances restrained to 0.84 (2) Å for O1, O3 and O4 of complex (I), and O13 and O14 of complex (II) yielding O—H distances of 0.748–0.828 Å. The remainder of the hydroxyl atoms were placed in calculated positions with O—H = 0.84 Å, and all *U*_iso_(H_OH_) were set to 1.5*U*_eq_(O).

## Supplementary Material

Crystal structure: contains datablock(s) I, II. DOI: 10.1107/S205698901502037X/lh5780sup1.cif

Structure factors: contains datablock(s) I. DOI: 10.1107/S205698901502037X/lh5780Isup2.hkl

Structure factors: contains datablock(s) II. DOI: 10.1107/S205698901502037X/lh5780IIsup3.hkl

CCDC references: 1433678, 1000450

Additional supporting information:  crystallographic information; 3D view; checkCIF report

## Figures and Tables

**Table 1:** Hydrogen-bond geometry (Å, °) for (I)

*D*—H⋯*A*	*D*—H	H⋯*A*	*D*⋯*A*	*D*—H⋯*A*
O5—H15⋯O11^i^	0.75 (4)	2.08 (4)	2.802 (4)	164 (4)
O1—H1⋯O6	0.83 (2)	1.78 (2)	2.583 (4)	163 (5)
O3—H3*B*⋯O5	0.83 (2)	1.70 (2)	2.510 (4)	164 (5)
O4—H4*B*⋯O2	0.83 (2)	1.73 (2)	2.528 (4)	161 (5)

Symmetry code: (i) 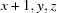.

**Table 2:** Hydrogen-bond geometry (Å, °) for (II)

*D*—H⋯*A*	*D*—H	H⋯*A*	*D*⋯*A*	*D*—H⋯*A*
O1—H1⋯O14	0.84	1.61	2.448 (3)	175
O2—H2*A*⋯O3	0.84	1.66	2.499 (3)	172
O5—H5⋯O13	0.84	1.70	2.536 (3)	170
O6—H6⋯O4	0.84	1.67	2.495 (3)	168
O13—H13*A*⋯O9^i^	0.83 (2)	1.98 (2)	2.763 (3)	158 (4)
O14—H14*A*⋯O9	0.82 (2)	1.94 (2)	2.738 (3)	163 (4)
O15—H15⋯O10^ii^	0.85 (5)	2.42 (5)	2.790 (4)	107 (4)

Symmetry codes: (i) 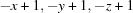; (ii) 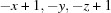.

**Table 3:** Experimental details

	(I)	(II)
Crystal data		
Chemical formula	[Cu_2_(C_10_H_7_N_2_O_2_)(OH)(NO_3_)_2_(C_10_H_8_N_2_O_2_)]	[Cu_6_(C_10_H_6_N_2_O_2_)_2_(CH_4_O)_2_(OH)_4_(NO_3_)_4_(C_10_H_8_N_2_O_2_)_4_]
*M*_r_	643.47	1886.53
Crystal system, space group	Triclinic, *P* 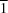	Triclinic, *P* 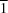
Temperature (K)	100	100
*a*, *b*, *c* (Å)	7.358 (2), 10.447 (3), 15.744 (4)	10.2135 (7), 13.3707 (8), 14.0565 (10)
α, β, γ (°)	77.610 (4), 78.927 (4), 69.938 (4)	64.591 (4), 75.659 (5), 82.262 (5)
*V* (Å^3^)	1101.1 (5)	1679.0 (2)
*Z*	2	1
Radiation type	Mo *K*α	Mo *K*α
μ (mm^−1^)	2.01	1.97
Crystal size (mm)	0.17 × 0.12 × 0.03	0.26 × 0.22 × 0.07

Data collection		
Diffractometer	Bruker SMART APEX CCD	Bruker APEXII CCD
Absorption correction	Multi-scan (*SADABS*; Bruker, 2009 ▸)	Multi-scan (*TWINABS*; Sheldrick, 2009 ▸)
*T*_min_, *T*_max_	0.605, 0.746	0.622, 0.746
No. of measured, independent and observed [*I* > 2σ(*I*)] reflections	14887, 6413, 4302	20719, 10920, 7847
*R*_int_	0.050	0.068
(sin θ/λ)_max_ (Å^−1^)	0.717	0.746

Refinement		
*R*[*F* ^2^ > 2σ(*F* ^2^)], *wR*(*F* ^2^), *S*	0.052, 0.125, 1.04	0.044, 0.119, 1.00
No. of reflections	6413	10920
No. of parameters	365	529
No. of restraints	3	2
H-atom treatment	H atoms treated by a mixture of independent and constrained refinement	H atoms treated by a mixture of independent and constrained refinement
Δρ_max_, Δρ_min_ (e Å^−3^)	1.11, −0.69	0.90, −1.08

Computer programs: *APEX2* and *SAINT* (Bruker, 2009 ▸, 2012 ▸), *SHELXS97* and *SHELXTL* (Sheldrick, 2008 ▸), *SHELXL2012* and *SHELXL2014/7* (Sheldrick, 2015 ▸), *SHELXLE* (Hübschle *et al.*, 2011 ▸) and *Mercury* (Macrae *et al.*, 2008 ▸).

## supplementary crystallographic information

### (I) (6,6'-Dihydroxybipyridine-2κ2N,N')[µ-6-(6-hydroxypyridin-2-yl)pyridin-2-olato-1:2κ3N,N':O2](µ-hydroxido-1:2κ2O:O')(µ-nitrato-1:2κ2O:O')(nitrato-1κO)dicopper(II) Crystal data

**Table:**

[Cu_2_(C_10_H_7_N_2_O_2_)(OH)(NO_3_)_2_(C_10_H_8_N_2_O_2_)]	*Z* = 2
*M**_r_* = 643.47	*F*(000) = 648
Triclinic, *P*1	*D*_x_ = 1.941 Mg m^−^^3^
*a* = 7.358 (2) Å	Mo *K*α radiation, λ = 0.71073 Å
*b* = 10.447 (3) Å	Cell parameters from 390 reflections
*c* = 15.744 (4) Å	θ = 2.3–24.8°
α = 77.610 (4)°	µ = 2.01 mm^−^^1^
β = 78.927 (4)°	*T* = 100 K
γ = 69.938 (4)°	Plate, green
*V* = 1101.1 (5) Å^3^	0.17 × 0.12 × 0.03 mm

### (I) (6,6'-Dihydroxybipyridine-2κ2N,N')[µ-6-(6-hydroxypyridin-2-yl)pyridin-2-olato-1:2κ3N,N':O2](µ-hydroxido-1:2κ2O:O')(µ-nitrato-1:2κ2O:O')(nitrato-1κO)dicopper(II) Data collection

**Table:**

Bruker SMART APEX CCD diffractometer	4302 reflections with *I* > 2σ(*I*)
Radiation source: fine-focus sealed tube	*R*_int_ = 0.050
ω scans	θ_max_ = 30.7°, θ_min_ = 1.3°
Absorption correction: multi-scan (SADABS; Bruker, 2009)	*h* = −10→10
*T*_min_ = 0.605, *T*_max_ = 0.746	*k* = −14→14
14887 measured reflections	*l* = −22→21
6413 independent reflections	

### (I) (6,6'-Dihydroxybipyridine-2κ2N,N')[µ-6-(6-hydroxypyridin-2-yl)pyridin-2-olato-1:2κ3N,N':O2](µ-hydroxido-1:2κ2O:O')(µ-nitrato-1:2κ2O:O')(nitrato-1κO)dicopper(II) Refinement

**Table:**

Refinement on *F*^2^	Primary atom site location: structure-invariant direct methods
Least-squares matrix: full	Secondary atom site location: difference Fourier map
*R*[*F*^2^ > 2σ(*F*^2^)] = 0.052	Hydrogen site location: mixed
*wR*(*F*^2^) = 0.125	H atoms treated by a mixture of independent and constrained refinement
*S* = 1.04	*w* = 1/[σ^2^(*F*_o_^2^) + (0.0555*P*)^2^ + 0.1969*P*] where *P* = (*F*_o_^2^ + 2*F*_c_^2^)/3
6413 reflections	(Δ/σ)_max_ < 0.001
365 parameters	Δρ_max_ = 1.11 e Å^−^^3^
3 restraints	Δρ_min_ = −0.69 e Å^−^^3^

### (I) (6,6'-Dihydroxybipyridine-2κ2N,N')[µ-6-(6-hydroxypyridin-2-yl)pyridin-2-olato-1:2κ3N,N':O2](µ-hydroxido-1:2κ2O:O')(µ-nitrato-1:2κ2O:O')(nitrato-1κO)dicopper(II) Special details

**Table:**

Geometry. All e.s.d.'s (except the e.s.d. in the dihedral angle between two l.s. planes) are estimated using the full covariance matrix. The cell e.s.d.'s are taken into account individually in the estimation of e.s.d.'s in distances, angles and torsion angles; correlations between e.s.d.'s in cell parameters are only used when they are defined by crystal symmetry. An approximate (isotropic) treatment of cell e.s.d.'s is used for estimating e.s.d.'s involving l.s. planes.

### (I) (6,6'-Dihydroxybipyridine-2κ2N,N')[µ-6-(6-hydroxypyridin-2-yl)pyridin-2-olato-1:2κ3N,N':O2](µ-hydroxido-1:2κ2O:O')(µ-nitrato-1:2κ2O:O')(nitrato-1κO)dicopper(II) Fractional atomic coordinates and isotropic or equivalent isotropic displacement parameters (Å^2^)

**Table:**

	*x*	*y*	*z*	*U*_iso_*/*U*_eq_	
C1	0.2710 (5)	0.4849 (4)	−0.0150 (3)	0.0185 (8)	
C2	0.2962 (6)	0.5280 (4)	−0.1049 (3)	0.0216 (8)	
H2	0.2206	0.6168	−0.1305	0.026*	
C3	0.4322 (6)	0.4401 (4)	−0.1562 (3)	0.0220 (8)	
H3	0.4521	0.4670	−0.2180	0.026*	
C4	0.5418 (5)	0.3099 (4)	−0.1166 (2)	0.0190 (8)	
H4	0.6379	0.2480	−0.1510	0.023*	
C5	0.5083 (5)	0.2731 (4)	−0.0271 (2)	0.0144 (7)	
C6	0.6100 (5)	0.1354 (4)	0.0196 (2)	0.0147 (7)	
C7	0.7447 (5)	0.0339 (4)	−0.0228 (3)	0.0190 (8)	
H7	0.7805	0.0511	−0.0845	0.023*	
C8	0.8294 (5)	−0.0964 (4)	0.0263 (3)	0.0187 (8)	
H8	0.9244	−0.1676	−0.0018	0.022*	
C9	0.7735 (5)	−0.1193 (4)	0.1146 (3)	0.0174 (8)	
H9	0.8290	−0.2065	0.1486	0.021*	
C10	0.6315 (5)	−0.0113 (4)	0.1552 (2)	0.0140 (7)	
C11	0.2728 (5)	0.2782 (4)	0.4525 (2)	0.0169 (7)	
C12	0.1854 (5)	0.3244 (4)	0.5324 (3)	0.0186 (8)	
H12	0.1660	0.4165	0.5392	0.022*	
C13	0.1285 (5)	0.2346 (4)	0.6005 (3)	0.0201 (8)	
H13	0.0705	0.2638	0.6553	0.024*	
C14	0.1560 (5)	0.0991 (4)	0.5894 (2)	0.0175 (7)	
H14	0.1177	0.0355	0.6362	0.021*	
C15	0.2398 (5)	0.0608 (4)	0.5090 (2)	0.0138 (7)	
H15	0.584 (6)	0.211 (4)	0.257 (3)	0.016 (12)*	
C16	0.2731 (5)	−0.0791 (4)	0.4908 (2)	0.0147 (7)	
C17	0.2193 (5)	−0.1803 (4)	0.5517 (2)	0.0164 (7)	
H17	0.1568	−0.1622	0.6085	0.020*	
C18	0.2577 (5)	−0.3093 (4)	0.5287 (2)	0.0172 (7)	
H18	0.2232	−0.3806	0.5702	0.021*	
C19	0.3449 (5)	−0.3331 (4)	0.4465 (3)	0.0191 (8)	
H19	0.3704	−0.4204	0.4299	0.023*	
C20	0.3961 (5)	−0.2269 (4)	0.3869 (2)	0.0172 (7)	
N1	0.3732 (4)	0.3613 (3)	0.0244 (2)	0.0156 (6)	
N2	0.5553 (4)	0.1137 (3)	0.10808 (19)	0.0129 (6)	
N3	0.2987 (4)	0.1489 (3)	0.44065 (19)	0.0126 (6)	
N4	0.3616 (4)	−0.1019 (3)	0.40843 (19)	0.0139 (6)	
N5	0.0443 (5)	0.4545 (4)	0.2519 (2)	0.0217 (7)	
N6	0.0449 (4)	0.1339 (3)	0.2131 (2)	0.0149 (6)	
O1	0.1390 (4)	0.5753 (3)	0.03228 (18)	0.0244 (6)	
H1	0.140 (7)	0.552 (5)	0.0859 (14)	0.037*	
O2	0.5740 (4)	−0.0383 (3)	0.23919 (17)	0.0181 (5)	
O3	0.3321 (4)	0.3654 (3)	0.38846 (17)	0.0194 (6)	
H3B	0.380 (6)	0.334 (5)	0.3421 (19)	0.029*	
O4	0.4801 (4)	−0.2525 (3)	0.30803 (18)	0.0217 (6)	
H4B	0.532 (6)	−0.195 (4)	0.280 (3)	0.033*	
O5	0.4755 (4)	0.2277 (3)	0.26553 (17)	0.0159 (5)	
O6	0.1872 (4)	0.4522 (3)	0.19101 (18)	0.0208 (6)	
O7	0.0328 (4)	0.3455 (3)	0.29893 (19)	0.0294 (7)	
O8	−0.0797 (4)	0.5672 (3)	0.2623 (2)	0.0367 (8)	
O9	0.1131 (4)	0.2090 (3)	0.15141 (17)	0.0205 (6)	
O10	0.1392 (4)	0.0637 (3)	0.2725 (2)	0.0290 (7)	
O11	−0.1223 (4)	0.1285 (3)	0.21196 (19)	0.0260 (7)	
Cu1	0.36658 (6)	0.27682 (5)	0.15407 (3)	0.01478 (12)	
Cu2	0.41333 (6)	0.06500 (5)	0.33097 (3)	0.01386 (12)	

### (I) (6,6'-Dihydroxybipyridine-2κ2N,N')[µ-6-(6-hydroxypyridin-2-yl)pyridin-2-olato-1:2κ3N,N':O2](µ-hydroxido-1:2κ2O:O')(µ-nitrato-1:2κ2O:O')(nitrato-1κO)dicopper(II) Atomic displacement parameters (Å^2^)

**Table:**

	*U*^11^	*U*^22^	*U*^33^	*U*^12^	*U*^13^	*U*^23^
C1	0.0167 (17)	0.0172 (19)	0.024 (2)	−0.0082 (15)	−0.0056 (15)	−0.0011 (16)
C2	0.0243 (19)	0.0138 (19)	0.027 (2)	−0.0079 (15)	−0.0079 (16)	0.0036 (16)
C3	0.027 (2)	0.023 (2)	0.0168 (19)	−0.0125 (17)	−0.0023 (15)	0.0030 (16)
C4	0.0224 (19)	0.020 (2)	0.0154 (18)	−0.0094 (16)	−0.0001 (15)	−0.0017 (15)
C5	0.0112 (15)	0.0162 (19)	0.0183 (18)	−0.0070 (14)	−0.0005 (13)	−0.0045 (15)
C6	0.0107 (15)	0.0180 (19)	0.0172 (18)	−0.0076 (14)	−0.0024 (13)	−0.0010 (15)
C7	0.0167 (17)	0.020 (2)	0.0198 (19)	−0.0075 (15)	0.0012 (14)	−0.0027 (16)
C8	0.0131 (16)	0.0170 (19)	0.025 (2)	−0.0026 (14)	−0.0009 (14)	−0.0059 (16)
C9	0.0140 (16)	0.0138 (18)	0.025 (2)	−0.0032 (14)	−0.0032 (14)	−0.0045 (15)
C10	0.0134 (16)	0.0148 (18)	0.0160 (18)	−0.0070 (14)	0.0003 (13)	−0.0045 (14)
C11	0.0169 (17)	0.0137 (18)	0.0212 (19)	−0.0045 (14)	−0.0071 (14)	−0.0013 (15)
C12	0.0181 (17)	0.0148 (19)	0.024 (2)	−0.0034 (14)	−0.0059 (15)	−0.0064 (16)
C13	0.0196 (18)	0.024 (2)	0.0180 (19)	−0.0063 (16)	−0.0011 (14)	−0.0077 (16)
C14	0.0155 (17)	0.019 (2)	0.0184 (18)	−0.0062 (15)	−0.0027 (14)	−0.0034 (15)
C15	0.0117 (15)	0.0145 (18)	0.0155 (17)	−0.0044 (13)	−0.0026 (13)	−0.0015 (14)
C16	0.0110 (15)	0.0150 (18)	0.0178 (18)	−0.0027 (13)	−0.0032 (13)	−0.0028 (14)
C17	0.0165 (17)	0.0191 (19)	0.0142 (17)	−0.0083 (15)	−0.0011 (13)	−0.0003 (15)
C18	0.0154 (17)	0.0164 (19)	0.0203 (19)	−0.0080 (14)	−0.0030 (14)	0.0014 (15)
C19	0.0198 (18)	0.0096 (18)	0.028 (2)	−0.0055 (14)	−0.0022 (15)	−0.0036 (15)
C20	0.0170 (17)	0.0154 (19)	0.0189 (19)	−0.0037 (14)	−0.0045 (14)	−0.0027 (15)
N1	0.0141 (14)	0.0139 (15)	0.0193 (16)	−0.0065 (12)	−0.0032 (12)	0.0006 (13)
N2	0.0117 (13)	0.0128 (15)	0.0139 (15)	−0.0040 (11)	−0.0006 (11)	−0.0025 (12)
N3	0.0120 (13)	0.0114 (15)	0.0143 (14)	−0.0028 (11)	−0.0021 (11)	−0.0030 (12)
N4	0.0126 (14)	0.0133 (15)	0.0156 (15)	−0.0035 (11)	−0.0029 (11)	−0.0020 (12)
N5	0.0220 (16)	0.0194 (18)	0.0257 (18)	−0.0022 (14)	−0.0097 (14)	−0.0092 (15)
N6	0.0140 (14)	0.0127 (15)	0.0178 (16)	−0.0039 (12)	0.0004 (12)	−0.0050 (13)
O1	0.0309 (15)	0.0146 (14)	0.0209 (15)	0.0007 (12)	−0.0024 (12)	−0.0024 (12)
O2	0.0208 (13)	0.0147 (13)	0.0151 (13)	−0.0051 (10)	0.0007 (10)	0.0016 (11)
O3	0.0295 (15)	0.0160 (14)	0.0154 (13)	−0.0103 (11)	−0.0021 (11)	−0.0036 (11)
O4	0.0325 (15)	0.0169 (14)	0.0171 (14)	−0.0110 (12)	0.0049 (11)	−0.0076 (11)
O5	0.0151 (13)	0.0151 (13)	0.0186 (14)	−0.0067 (11)	−0.0021 (10)	−0.0019 (11)
O6	0.0240 (14)	0.0142 (14)	0.0225 (14)	−0.0041 (11)	−0.0024 (11)	−0.0027 (11)
O7	0.0319 (16)	0.0290 (17)	0.0274 (16)	−0.0131 (13)	0.0039 (13)	−0.0061 (14)
O8	0.0320 (17)	0.0305 (18)	0.0410 (19)	0.0030 (14)	0.0008 (14)	−0.0180 (15)
O9	0.0215 (13)	0.0256 (15)	0.0160 (13)	−0.0125 (12)	0.0007 (10)	−0.0013 (12)
O10	0.0244 (15)	0.0310 (17)	0.0330 (17)	−0.0126 (13)	−0.0151 (13)	0.0083 (14)
O11	0.0140 (13)	0.0296 (16)	0.0354 (17)	−0.0104 (12)	−0.0033 (12)	−0.0012 (14)
Cu1	0.0146 (2)	0.0141 (2)	0.0145 (2)	−0.00367 (17)	−0.00074 (16)	−0.00225 (18)
Cu2	0.0155 (2)	0.0128 (2)	0.0137 (2)	−0.00583 (17)	−0.00062 (16)	−0.00191 (17)

### (I) (6,6'-Dihydroxybipyridine-2κ2N,N')[µ-6-(6-hydroxypyridin-2-yl)pyridin-2-olato-1:2κ3N,N':O2](µ-hydroxido-1:2κ2O:O')(µ-nitrato-1:2κ2O:O')(nitrato-1κO)dicopper(II) Geometric parameters (Å, º)

**Table:**

C1—O1	1.335 (5)	C15—C16	1.478 (5)
C1—N1	1.336 (5)	C16—N4	1.366 (4)
C1—C2	1.388 (6)	C16—C17	1.378 (5)
C2—C3	1.369 (6)	C17—C18	1.391 (5)
C2—H2	0.9500	C17—H17	0.9500
C3—C4	1.402 (5)	C18—C19	1.363 (5)
C3—H3	0.9500	C18—H18	0.9500
C4—C5	1.376 (5)	C19—C20	1.399 (5)
C4—H4	0.9500	C19—H19	0.9500
C5—N1	1.370 (4)	C20—O4	1.316 (4)
C5—C6	1.480 (5)	C20—N4	1.346 (5)
C6—N2	1.366 (5)	N1—Cu1	2.042 (3)
C6—C7	1.372 (5)	N2—Cu1	1.969 (3)
C7—C8	1.411 (5)	N3—Cu2	2.011 (3)
C7—H7	0.9500	N4—Cu2	2.009 (3)
C8—C9	1.366 (5)	N5—O8	1.238 (4)
C8—H8	0.9500	N5—O7	1.239 (4)
C9—C10	1.420 (5)	N5—O6	1.276 (4)
C9—H9	0.9500	N6—O10	1.225 (4)
C10—O2	1.310 (4)	N6—O9	1.251 (4)
C10—N2	1.346 (5)	N6—O11	1.254 (4)
C11—O3	1.322 (5)	O1—H1	0.827 (19)
C11—N3	1.347 (5)	O2—Cu2	1.946 (3)
C11—C12	1.402 (5)	O3—H3B	0.829 (19)
C12—C13	1.367 (5)	O4—H4B	0.827 (19)
C12—H12	0.9500	O5—Cu2	1.939 (3)
C13—C14	1.404 (5)	O5—Cu1	1.964 (3)
C13—H13	0.9500	O5—H15	0.75 (4)
C14—C15	1.374 (5)	O6—Cu1	1.985 (3)
C14—H14	0.9500	O9—Cu1	2.221 (3)
C15—N3	1.365 (5)	O10—Cu2	2.377 (3)
			
O1—C1—N1	120.4 (3)	C18—C19—C20	119.0 (4)
O1—C1—C2	116.4 (3)	C18—C19—H19	120.5
N1—C1—C2	123.2 (4)	C20—C19—H19	120.5
C3—C2—C1	118.7 (4)	O4—C20—N4	120.3 (3)
C3—C2—H2	120.7	O4—C20—C19	117.8 (3)
C1—C2—H2	120.7	N4—C20—C19	121.9 (3)
C2—C3—C4	119.3 (4)	C1—N1—C5	118.0 (3)
C2—C3—H3	120.3	C1—N1—Cu1	130.4 (3)
C4—C3—H3	120.3	C5—N1—Cu1	111.6 (2)
C5—C4—C3	119.1 (4)	C10—N2—C6	119.6 (3)
C5—C4—H4	120.5	C10—N2—Cu1	126.3 (2)
C3—C4—H4	120.5	C6—N2—Cu1	114.1 (2)
N1—C5—C4	121.7 (3)	C11—N3—C15	118.6 (3)
N1—C5—C6	115.6 (3)	C11—N3—Cu2	127.8 (2)
C4—C5—C6	122.7 (3)	C15—N3—Cu2	113.6 (2)
N2—C6—C7	121.8 (3)	C20—N4—C16	118.6 (3)
N2—C6—C5	115.4 (3)	C20—N4—Cu2	127.7 (2)
C7—C6—C5	122.7 (3)	C16—N4—Cu2	113.7 (2)
C6—C7—C8	119.1 (3)	O8—N5—O7	121.6 (4)
C6—C7—H7	120.4	O8—N5—O6	118.6 (4)
C8—C7—H7	120.4	O7—N5—O6	119.8 (3)
C9—C8—C7	119.4 (3)	O10—N6—O9	121.6 (3)
C9—C8—H8	120.3	O10—N6—O11	119.9 (3)
C7—C8—H8	120.3	O9—N6—O11	118.5 (3)
C8—C9—C10	119.3 (4)	C1—O1—H1	114 (3)
C8—C9—H9	120.4	C10—O2—Cu2	137.1 (2)
C10—C9—H9	120.4	C11—O3—H3B	114 (3)
O2—C10—N2	121.2 (3)	C20—O4—H4B	114 (3)
O2—C10—C9	117.9 (3)	Cu2—O5—Cu1	108.02 (13)
N2—C10—C9	120.9 (3)	Cu2—O5—H15	108 (3)
O3—C11—N3	120.3 (3)	Cu1—O5—H15	110 (3)
O3—C11—C12	117.9 (3)	N5—O6—Cu1	121.9 (2)
N3—C11—C12	121.8 (3)	N6—O9—Cu1	124.9 (2)
C13—C12—C11	118.9 (4)	N6—O10—Cu2	135.9 (2)
C13—C12—H12	120.5	O5—Cu1—N2	92.97 (12)
C11—C12—H12	120.5	O5—Cu1—O6	89.59 (11)
C12—C13—C14	119.9 (4)	N2—Cu1—O6	174.53 (12)
C12—C13—H13	120.0	O5—Cu1—N1	150.90 (12)
C14—C13—H13	120.0	N2—Cu1—N1	82.98 (12)
C15—C14—C13	118.3 (4)	O6—Cu1—N1	92.44 (12)
C15—C14—H14	120.8	O5—Cu1—O9	116.83 (10)
C13—C14—H14	120.8	N2—Cu1—O9	92.96 (11)
N3—C15—C14	122.4 (3)	O6—Cu1—O9	90.19 (11)
N3—C15—C16	115.4 (3)	N1—Cu1—O9	92.20 (11)
C14—C15—C16	122.2 (3)	O5—Cu2—O2	89.00 (11)
N4—C16—C17	121.7 (3)	O5—Cu2—N4	174.31 (12)
N4—C16—C15	115.1 (3)	O2—Cu2—N4	93.75 (11)
C17—C16—C15	123.2 (3)	O5—Cu2—N3	94.05 (12)
C16—C17—C18	119.0 (3)	O2—Cu2—N3	167.14 (11)
C16—C17—H17	120.5	N4—Cu2—N3	82.20 (12)
C18—C17—H17	120.5	O5—Cu2—O10	104.96 (11)
C19—C18—C17	119.8 (3)	O2—Cu2—O10	86.67 (11)
C19—C18—H18	120.1	N4—Cu2—O10	80.20 (11)
C17—C18—H18	120.1	N3—Cu2—O10	104.54 (11)
			
O1—C1—C2—C3	178.8 (3)	C4—C5—N1—C1	1.1 (5)
N1—C1—C2—C3	0.0 (6)	C6—C5—N1—C1	−177.1 (3)
C1—C2—C3—C4	−0.2 (6)	C4—C5—N1—Cu1	−178.2 (3)
C2—C3—C4—C5	0.7 (6)	C6—C5—N1—Cu1	3.6 (4)
C3—C4—C5—N1	−1.2 (5)	O2—C10—N2—C6	−176.0 (3)
C3—C4—C5—C6	176.9 (3)	C9—C10—N2—C6	2.4 (5)
N1—C5—C6—N2	0.3 (5)	O2—C10—N2—Cu1	4.4 (5)
C4—C5—C6—N2	−177.8 (3)	C9—C10—N2—Cu1	−177.2 (3)
N1—C5—C6—C7	177.9 (3)	C7—C6—N2—C10	−1.5 (5)
C4—C5—C6—C7	−0.3 (6)	C5—C6—N2—C10	176.0 (3)
N2—C6—C7—C8	−0.2 (5)	C7—C6—N2—Cu1	178.1 (3)
C5—C6—C7—C8	−177.5 (3)	C5—C6—N2—Cu1	−4.3 (4)
C6—C7—C8—C9	1.0 (5)	O3—C11—N3—C15	178.4 (3)
C7—C8—C9—C10	−0.1 (5)	C12—C11—N3—C15	−0.8 (5)
C8—C9—C10—O2	176.8 (3)	O3—C11—N3—Cu2	−2.6 (5)
C8—C9—C10—N2	−1.6 (5)	C12—C11—N3—Cu2	178.2 (3)
O3—C11—C12—C13	−177.9 (3)	C14—C15—N3—C11	−0.3 (5)
N3—C11—C12—C13	1.3 (5)	C16—C15—N3—C11	180.0 (3)
C11—C12—C13—C14	−0.7 (6)	C14—C15—N3—Cu2	−179.4 (3)
C12—C13—C14—C15	−0.3 (5)	C16—C15—N3—Cu2	0.9 (4)
C13—C14—C15—N3	0.8 (5)	O4—C20—N4—C16	179.6 (3)
C13—C14—C15—C16	−179.5 (3)	C19—C20—N4—C16	−0.3 (5)
N3—C15—C16—N4	1.1 (4)	O4—C20—N4—Cu2	2.8 (5)
C14—C15—C16—N4	−178.6 (3)	C19—C20—N4—Cu2	−177.1 (3)
N3—C15—C16—C17	−178.7 (3)	C17—C16—N4—C20	0.0 (5)
C14—C15—C16—C17	1.6 (5)	C15—C16—N4—C20	−179.8 (3)
N4—C16—C17—C18	0.6 (5)	C17—C16—N4—Cu2	177.3 (3)
C15—C16—C17—C18	−179.6 (3)	C15—C16—N4—Cu2	−2.5 (4)
C16—C17—C18—C19	−0.9 (5)	N2—C10—O2—Cu2	−9.7 (5)
C17—C18—C19—C20	0.7 (6)	C9—C10—O2—Cu2	171.9 (3)
C18—C19—C20—O4	180.0 (3)	O8—N5—O6—Cu1	−168.6 (3)
C18—C19—C20—N4	−0.1 (6)	O7—N5—O6—Cu1	11.6 (5)
O1—C1—N1—C5	−179.2 (3)	O10—N6—O9—Cu1	−18.1 (5)
C2—C1—N1—C5	−0.4 (5)	O11—N6—O9—Cu1	163.9 (2)
O1—C1—N1—Cu1	−0.2 (5)	O9—N6—O10—Cu2	20.0 (5)
C2—C1—N1—Cu1	178.6 (3)	O11—N6—O10—Cu2	−162.0 (3)

### (I) (6,6'-Dihydroxybipyridine-2κ2N,N')[µ-6-(6-hydroxypyridin-2-yl)pyridin-2-olato-1:2κ3N,N':O2](µ-hydroxido-1:2κ2O:O')(µ-nitrato-1:2κ2O:O')(nitrato-1κO)dicopper(II) Hydrogen-bond geometry (Å, º)

**Table:**

*D*—H···*A*	*D*—H	H···*A*	*D*···*A*	*D*—H···*A*
O5—H15···O11^i^	0.75 (4)	2.08 (4)	2.802 (4)	164 (4)
O1—H1···O6	0.83 (2)	1.78 (2)	2.583 (4)	163 (5)
O3—H3*B*···O5	0.83 (2)	1.70 (2)	2.510 (4)	164 (5)
O4—H4*B*···O2	0.83 (2)	1.73 (2)	2.528 (4)	161 (5)

Symmetry code: (i) *x*+1, *y*, *z*.

### (II) Bis(µ3-bipyridine-2,2'-diolato-κ3O:N,N':O')tetrakis(6,6'-dihydroxybipyridine-κ2N,N')tetrakis(µ-hydroxido-κ2O:O')bis(methanol-κO)tetrakis(µ-nitrato-κ2O:O')hexacopper(II) Crystal data

**Table:**

[Cu_6_(C_10_H_6_N_2_O_2_)_2_(CH_4_O)_2_(OH)_4_(NO_3_)_4_(C_10_H_8_N_2_O_2_)_4_]	*Z* = 1
*M**_r_* = 1886.53	*F*(000) = 954
Triclinic, *P*1	*D*_x_ = 1.866 Mg m^−^^3^
*a* = 10.2135 (7) Å	Mo *K*α radiation, λ = 0.71073 Å
*b* = 13.3707 (8) Å	Cell parameters from 1117 reflections
*c* = 14.0565 (10) Å	θ = 3.0–29.1°
α = 64.591 (4)°	µ = 1.97 mm^−^^1^
β = 75.659 (5)°	*T* = 100 K
γ = 82.262 (5)°	Prism, green
*V* = 1679.0 (2) Å^3^	0.26 × 0.22 × 0.07 mm

### (II) Bis(µ3-bipyridine-2,2'-diolato-κ3O:N,N':O')tetrakis(6,6'-dihydroxybipyridine-κ2N,N')tetrakis(µ-hydroxido-κ2O:O')bis(methanol-κO)tetrakis(µ-nitrato-κ2O:O')hexacopper(II) Data collection

**Table:**

Bruker APEXII CCD diffractometer	7847 reflections with *I* > 2σ(*I*)
φ and ω scans	*R*_int_ = 0.068
Absorption correction: multi-scan (*TWINABS*; Sheldrick, 2009)	θ_max_ = 32.0°, θ_min_ = 1.6°
*T*_min_ = 0.622, *T*_max_ = 0.746	*h* = −14→14
20719 measured reflections	*k* = −16→19
10920 independent reflections	*l* = 0→20

### (II) Bis(µ3-bipyridine-2,2'-diolato-κ3O:N,N':O')tetrakis(6,6'-dihydroxybipyridine-κ2N,N')tetrakis(µ-hydroxido-κ2O:O')bis(methanol-κO)tetrakis(µ-nitrato-κ2O:O')hexacopper(II) Refinement

**Table:**

Refinement on *F*^2^	Primary atom site location: structure-invariant direct methods
Least-squares matrix: full	Secondary atom site location: difference Fourier map
*R*[*F*^2^ > 2σ(*F*^2^)] = 0.044	Hydrogen site location: mixed
*wR*(*F*^2^) = 0.119	H atoms treated by a mixture of independent and constrained refinement
*S* = 1.00	*w* = 1/[σ^2^(*F*_o_^2^) + (0.068*P*)^2^] where *P* = (*F*_o_^2^ + 2*F*_c_^2^)/3
10920 reflections	(Δ/σ)_max_ = 0.001
529 parameters	Δρ_max_ = 0.90 e Å^−^^3^
2 restraints	Δρ_min_ = −1.08 e Å^−^^3^

### (II) Bis(µ3-bipyridine-2,2'-diolato-κ3O:N,N':O')tetrakis(6,6'-dihydroxybipyridine-κ2N,N')tetrakis(µ-hydroxido-κ2O:O')bis(methanol-κO)tetrakis(µ-nitrato-κ2O:O')hexacopper(II) Special details

**Table:**

Experimental. The crystal under investigation was found to be split with two major domains not related by any obvious twin operation. The orientation matrices for the two components were identified using the program Cell_Now, with the two components being related by a 2.9 degrees rotation about either the reciprocal axis 1.000 - 0.363 - 0.339 or the real axis 1.000 - 0.178 - 0.265 degree. The two components were integrated using Saint, resulting in in the following statistics:17535 data (5769 unique) involve domain 1 only, mean I/sigma 8.6 17271 data (5689 unique) involve domain 2 only, mean I/sigma 8.2 34813 data (9811 unique) involve 2 domains, mean I/sigma 9.5 11 data (11 unique) involve 3 domains, mean I/sigma 8.7 4 data (2 unique) involve 4 domains, mean I/sigma 57.6The exact correlation matrix was identified by the integration program was found to beTransforms h1.1(1)->h1.2(2) 1.00336 0.02923 - 0.02720 - 0.01894 1.02272 - 0.04903 0.02520 0.05747 0.97055.The data were corrected for absorption using twinabs, and the structure was solved using direct methods with only the non-overlapping reflections of component 1. The structure was refined using the hklf 5 routine with all reflections of component 1 (including the overlapping ones), resulting in a BASF value of 0.486 (1).The *R*_int_ value given is for all reflections and is based on agreement between observed single and composite intensities and those calculated from refined unique intensities and twin fractions (TWINABS (Sheldrick, 2009)).
Geometry. All e.s.d.'s (except the e.s.d. in the dihedral angle between two l.s. planes) are estimated using the full covariance matrix. The cell e.s.d.'s are taken into account individually in the estimation of e.s.d.'s in distances, angles and torsion angles; correlations between e.s.d.'s in cell parameters are only used when they are defined by crystal symmetry. An approximate (isotropic) treatment of cell e.s.d.'s is used for estimating e.s.d.'s involving l.s. planes.
Refinement. Refined as a 2-component twin.

### (II) Bis(µ3-bipyridine-2,2'-diolato-κ3O:N,N':O')tetrakis(6,6'-dihydroxybipyridine-κ2N,N')tetrakis(µ-hydroxido-κ2O:O')bis(methanol-κO)tetrakis(µ-nitrato-κ2O:O')hexacopper(II) Fractional atomic coordinates and isotropic or equivalent isotropic displacement parameters (Å^2^)

**Table:**

	*x*	*y*	*z*	*U*_iso_*/*U*_eq_	
Cu1	0.41028 (4)	0.22335 (3)	0.75942 (3)	0.01004 (9)	
Cu2	0.48719 (4)	0.28424 (3)	0.49234 (3)	0.01009 (9)	
Cu3	0.79681 (4)	0.28550 (3)	0.32300 (3)	0.00978 (9)	
O1	0.6858 (2)	0.3553 (2)	0.66647 (18)	0.0149 (5)	
H1	0.6187	0.3498	0.6447	0.022*	
O2	0.1351 (2)	0.0820 (2)	0.89259 (19)	0.0210 (6)	
H2A	0.1769	0.1133	0.8284	0.031*	
O3	0.2766 (2)	0.17719 (19)	0.70803 (17)	0.0108 (4)	
O4	0.6689 (2)	0.3846 (2)	0.23790 (17)	0.0154 (5)	
O5	0.8343 (2)	0.1430 (2)	0.56936 (18)	0.0162 (5)	
H5	0.7765	0.1877	0.5382	0.024*	
O6	0.8053 (2)	0.3901 (2)	0.06196 (18)	0.0174 (5)	
H6	0.7504	0.3859	0.1189	0.026*	
O7	0.2336 (2)	0.3684 (2)	0.77329 (18)	0.0169 (5)	
O8	0.1064 (2)	0.5167 (2)	0.71865 (19)	0.0172 (5)	
O9	0.2815 (2)	0.49085 (19)	0.60670 (18)	0.0166 (5)	
O10	0.5101 (3)	−0.0537 (2)	0.6858 (2)	0.0244 (6)	
O11	0.5594 (2)	0.0977 (2)	0.68885 (19)	0.0160 (5)	
O12	0.4932 (2)	0.1038 (2)	0.55029 (19)	0.0164 (5)	
O13	0.6824 (2)	0.28679 (18)	0.45612 (17)	0.0103 (4)	
H13A	0.695 (4)	0.347 (2)	0.455 (3)	0.015*	
O14	0.4843 (2)	0.33310 (19)	0.61349 (17)	0.0105 (4)	
H14A	0.432 (3)	0.387 (2)	0.598 (3)	0.016*	
O15	0.7227 (3)	0.1269 (2)	0.3314 (2)	0.0262 (6)	
H15	0.647 (4)	0.121 (4)	0.375 (4)	0.039*	
N1	0.5541 (2)	0.2397 (2)	0.8244 (2)	0.0105 (5)	
N2	0.3295 (3)	0.1288 (2)	0.9159 (2)	0.0111 (5)	
N3	0.2886 (2)	0.2882 (2)	0.5293 (2)	0.0104 (5)	
N4	0.4511 (2)	0.3683 (2)	0.3394 (2)	0.0102 (5)	
N5	0.9518 (3)	0.2047 (2)	0.3927 (2)	0.0110 (5)	
N6	0.9403 (3)	0.3065 (2)	0.1862 (2)	0.0108 (5)	
N7	0.2064 (3)	0.4580 (2)	0.7012 (2)	0.0125 (5)	
N8	0.5208 (3)	0.0495 (2)	0.6423 (2)	0.0134 (6)	
C1	0.6682 (3)	0.2959 (3)	0.7701 (3)	0.0132 (6)	
C2	0.7689 (3)	0.2919 (3)	0.8239 (3)	0.0148 (7)	
H2	0.8502	0.3303	0.7848	0.018*	
C3	0.7495 (3)	0.2321 (3)	0.9335 (3)	0.0174 (7)	
H3	0.8176	0.2287	0.9703	0.021*	
C4	0.6293 (3)	0.1761 (3)	0.9908 (3)	0.0168 (7)	
H4	0.6131	0.1359	1.0668	0.020*	
C5	0.5354 (3)	0.1810 (3)	0.9336 (3)	0.0115 (6)	
C6	0.4062 (3)	0.1227 (3)	0.9856 (3)	0.0124 (7)	
C7	0.3656 (3)	0.0679 (3)	1.0958 (3)	0.0157 (7)	
H7	0.4210	0.0644	1.1423	0.019*	
C8	0.2402 (3)	0.0176 (3)	1.1371 (3)	0.0177 (7)	
H8	0.2100	−0.0214	1.2127	0.021*	
C9	0.1613 (3)	0.0243 (3)	1.0695 (3)	0.0173 (7)	
H9	0.0750	−0.0079	1.0971	0.021*	
C10	0.2101 (3)	0.0798 (3)	0.9580 (3)	0.0148 (7)	
C11	0.2146 (3)	0.2432 (3)	0.6310 (3)	0.0118 (6)	
C12	0.0747 (3)	0.2651 (3)	0.6532 (3)	0.0147 (7)	
H12	0.0230	0.2328	0.7248	0.018*	
C13	0.0135 (3)	0.3340 (3)	0.5699 (3)	0.0162 (7)	
H13	−0.0806	0.3515	0.5841	0.019*	
C14	0.0893 (3)	0.3782 (3)	0.4647 (3)	0.0134 (6)	
H14	0.0480	0.4249	0.4063	0.016*	
C15	0.2260 (3)	0.3523 (3)	0.4478 (3)	0.0107 (6)	
C16	0.3161 (3)	0.3916 (3)	0.3392 (3)	0.0107 (6)	
C17	0.2675 (3)	0.4448 (3)	0.2472 (3)	0.0136 (7)	
H17	0.1728	0.4556	0.2506	0.016*	
C18	0.3584 (3)	0.4833 (3)	0.1480 (3)	0.0170 (7)	
H18	0.3268	0.5215	0.0829	0.020*	
C19	0.4939 (3)	0.4648 (3)	0.1461 (3)	0.0155 (7)	
H19	0.5575	0.4926	0.0795	0.019*	
C20	0.5395 (3)	0.4043 (3)	0.2436 (2)	0.0124 (6)	
C21	0.9476 (3)	0.1470 (3)	0.4981 (3)	0.0132 (6)	
C22	1.0618 (3)	0.0891 (3)	0.5372 (3)	0.0150 (7)	
H22	1.0561	0.0473	0.6124	0.018*	
C23	1.1811 (3)	0.0938 (3)	0.4656 (3)	0.0163 (7)	
H23	1.2598	0.0562	0.4906	0.020*	
C24	1.1870 (3)	0.1542 (3)	0.3551 (3)	0.0152 (7)	
H24	1.2693	0.1577	0.3044	0.018*	
C25	1.0716 (3)	0.2084 (3)	0.3209 (3)	0.0120 (6)	
C26	1.0664 (3)	0.2698 (3)	0.2063 (3)	0.0121 (6)	
C27	1.1783 (3)	0.2865 (3)	0.1232 (3)	0.0169 (7)	
H27	1.2658	0.2639	0.1385	0.020*	
C28	1.1625 (3)	0.3365 (3)	0.0176 (3)	0.0175 (7)	
H28	1.2391	0.3471	−0.0398	0.021*	
C29	1.0370 (3)	0.3706 (3)	−0.0045 (3)	0.0165 (7)	
H29	1.0243	0.4034	−0.0765	0.020*	
C30	0.9276 (3)	0.3557 (3)	0.0827 (3)	0.0136 (7)	
C31	0.7846 (4)	0.0935 (4)	0.2443 (3)	0.0308 (9)	
H31A	0.8829	0.1003	0.2282	0.046*	
H31B	0.7492	0.1413	0.1800	0.046*	
H31C	0.7636	0.0164	0.2659	0.046*	

### (II) Bis(µ3-bipyridine-2,2'-diolato-κ3O:N,N':O')tetrakis(6,6'-dihydroxybipyridine-κ2N,N')tetrakis(µ-hydroxido-κ2O:O')bis(methanol-κO)tetrakis(µ-nitrato-κ2O:O')hexacopper(II) Atomic displacement parameters (Å^2^)

**Table:**

	*U*^11^	*U*^22^	*U*^33^	*U*^12^	*U*^13^	*U*^23^
Cu1	0.00874 (18)	0.0129 (2)	0.00790 (18)	−0.00220 (14)	−0.00128 (13)	−0.00351 (15)
Cu2	0.00726 (17)	0.0145 (2)	0.00810 (18)	−0.00063 (13)	−0.00178 (13)	−0.00408 (15)
Cu3	0.00937 (18)	0.0123 (2)	0.00782 (18)	0.00178 (14)	−0.00247 (13)	−0.00451 (15)
O1	0.0088 (10)	0.0228 (14)	0.0133 (11)	−0.0060 (9)	−0.0021 (8)	−0.0062 (10)
O2	0.0149 (12)	0.0354 (17)	0.0115 (12)	−0.0138 (11)	−0.0006 (9)	−0.0061 (12)
O3	0.0098 (10)	0.0145 (12)	0.0072 (10)	−0.0033 (8)	−0.0019 (8)	−0.0028 (9)
O4	0.0101 (11)	0.0237 (14)	0.0083 (10)	0.0025 (9)	−0.0031 (8)	−0.0030 (10)
O5	0.0126 (11)	0.0195 (13)	0.0129 (11)	0.0018 (9)	−0.0040 (9)	−0.0033 (10)
O6	0.0135 (11)	0.0266 (15)	0.0115 (11)	0.0047 (10)	−0.0033 (9)	−0.0084 (11)
O7	0.0218 (12)	0.0130 (13)	0.0113 (11)	0.0030 (9)	−0.0016 (9)	−0.0027 (10)
O8	0.0127 (11)	0.0189 (13)	0.0217 (13)	0.0050 (9)	−0.0035 (9)	−0.0115 (11)
O9	0.0205 (12)	0.0140 (12)	0.0109 (11)	0.0007 (9)	0.0025 (9)	−0.0046 (10)
O10	0.0251 (13)	0.0103 (13)	0.0334 (15)	−0.0018 (10)	−0.0150 (11)	0.0000 (12)
O11	0.0130 (11)	0.0183 (13)	0.0194 (12)	−0.0009 (9)	−0.0042 (9)	−0.0098 (11)
O12	0.0171 (12)	0.0158 (13)	0.0159 (12)	0.0013 (9)	−0.0061 (9)	−0.0051 (10)
O13	0.0088 (10)	0.0124 (12)	0.0098 (10)	0.0007 (8)	−0.0029 (8)	−0.0047 (9)
O14	0.0096 (10)	0.0110 (11)	0.0111 (10)	0.0008 (8)	−0.0029 (8)	−0.0046 (9)
O15	0.0306 (15)	0.0195 (15)	0.0308 (16)	−0.0048 (12)	−0.0132 (12)	−0.0076 (13)
N1	0.0096 (12)	0.0133 (14)	0.0089 (12)	0.0012 (10)	−0.0016 (10)	−0.0055 (11)
N2	0.0104 (12)	0.0113 (14)	0.0097 (12)	−0.0029 (10)	−0.0018 (10)	−0.0022 (11)
N3	0.0096 (12)	0.0122 (14)	0.0117 (13)	−0.0002 (9)	−0.0020 (10)	−0.0074 (11)
N4	0.0100 (12)	0.0109 (13)	0.0103 (12)	0.0008 (10)	−0.0044 (10)	−0.0042 (11)
N5	0.0111 (12)	0.0104 (13)	0.0115 (13)	0.0007 (10)	−0.0023 (10)	−0.0049 (11)
N6	0.0130 (13)	0.0100 (14)	0.0099 (12)	0.0015 (10)	−0.0026 (10)	−0.0051 (11)
N7	0.0151 (13)	0.0130 (14)	0.0111 (13)	−0.0017 (10)	−0.0009 (10)	−0.0073 (11)
N8	0.0107 (13)	0.0095 (14)	0.0179 (14)	−0.0001 (10)	−0.0046 (11)	−0.0029 (12)
C1	0.0140 (15)	0.0133 (17)	0.0138 (15)	0.0006 (12)	−0.0024 (12)	−0.0077 (13)
C2	0.0085 (14)	0.0195 (19)	0.0196 (17)	−0.0022 (12)	−0.0028 (12)	−0.0108 (15)
C3	0.0151 (16)	0.0193 (19)	0.0204 (18)	−0.0002 (13)	−0.0081 (13)	−0.0085 (15)
C4	0.0187 (17)	0.021 (2)	0.0120 (15)	0.0006 (14)	−0.0055 (13)	−0.0075 (14)
C5	0.0102 (14)	0.0146 (17)	0.0126 (15)	0.0018 (12)	−0.0039 (12)	−0.0082 (13)
C6	0.0125 (15)	0.0115 (17)	0.0118 (15)	0.0019 (12)	−0.0033 (12)	−0.0039 (13)
C7	0.0185 (17)	0.0177 (19)	0.0101 (15)	0.0015 (13)	−0.0032 (12)	−0.0055 (14)
C8	0.0225 (18)	0.0147 (18)	0.0115 (15)	−0.0025 (13)	0.0008 (13)	−0.0031 (14)
C9	0.0147 (16)	0.020 (2)	0.0141 (16)	−0.0062 (13)	0.0032 (13)	−0.0059 (15)
C10	0.0126 (15)	0.0181 (18)	0.0141 (16)	−0.0011 (13)	−0.0026 (12)	−0.0069 (14)
C11	0.0106 (14)	0.0118 (16)	0.0122 (15)	−0.0034 (11)	−0.0001 (11)	−0.0045 (13)
C12	0.0116 (15)	0.0191 (18)	0.0152 (16)	−0.0013 (12)	−0.0005 (12)	−0.0097 (14)
C13	0.0102 (14)	0.0206 (18)	0.0197 (17)	0.0006 (12)	−0.0013 (12)	−0.0113 (15)
C14	0.0130 (15)	0.0147 (17)	0.0136 (15)	0.0017 (12)	−0.0051 (12)	−0.0061 (13)
C15	0.0101 (14)	0.0117 (16)	0.0123 (15)	−0.0001 (11)	−0.0043 (11)	−0.0059 (13)
C16	0.0118 (14)	0.0093 (15)	0.0136 (15)	0.0012 (11)	−0.0039 (11)	−0.0069 (13)
C17	0.0139 (15)	0.0135 (17)	0.0148 (16)	0.0030 (12)	−0.0055 (12)	−0.0067 (14)
C18	0.0197 (17)	0.021 (2)	0.0119 (15)	0.0066 (14)	−0.0076 (13)	−0.0075 (14)
C19	0.0161 (16)	0.0209 (19)	0.0069 (14)	0.0047 (13)	−0.0035 (12)	−0.0042 (14)
C20	0.0129 (15)	0.0151 (17)	0.0091 (14)	0.0026 (12)	−0.0034 (11)	−0.0051 (13)
C21	0.0148 (15)	0.0116 (16)	0.0143 (15)	−0.0009 (12)	−0.0033 (12)	−0.0059 (13)
C22	0.0175 (16)	0.0135 (17)	0.0139 (15)	0.0004 (12)	−0.0079 (12)	−0.0035 (13)
C23	0.0151 (16)	0.0149 (17)	0.0189 (17)	0.0051 (12)	−0.0077 (13)	−0.0063 (14)
C24	0.0112 (15)	0.0180 (18)	0.0194 (17)	0.0028 (12)	−0.0036 (12)	−0.0112 (15)
C25	0.0128 (15)	0.0114 (16)	0.0138 (15)	0.0025 (11)	−0.0025 (12)	−0.0083 (13)
C26	0.0125 (15)	0.0116 (16)	0.0167 (16)	0.0014 (12)	−0.0053 (12)	−0.0093 (13)
C27	0.0137 (16)	0.0205 (19)	0.0196 (17)	0.0022 (13)	−0.0031 (13)	−0.0121 (15)
C28	0.0150 (16)	0.021 (2)	0.0174 (16)	−0.0005 (13)	0.0017 (13)	−0.0118 (15)
C29	0.0191 (17)	0.0209 (19)	0.0107 (15)	0.0016 (14)	−0.0031 (12)	−0.0083 (14)
C30	0.0149 (16)	0.0116 (17)	0.0136 (15)	0.0032 (12)	−0.0021 (12)	−0.0060 (13)
C31	0.036 (2)	0.030 (2)	0.032 (2)	−0.0003 (18)	−0.0100 (18)	−0.016 (2)

### (II) Bis(µ3-bipyridine-2,2'-diolato-κ3O:N,N':O')tetrakis(6,6'-dihydroxybipyridine-κ2N,N')tetrakis(µ-hydroxido-κ2O:O')bis(methanol-κO)tetrakis(µ-nitrato-κ2O:O')hexacopper(II) Geometric parameters (Å, º)

**Table:**

Cu1—O3	1.966 (2)	C1—C2	1.403 (5)
Cu1—O14	1.970 (2)	C2—C3	1.373 (5)
Cu1—N1	1.991 (3)	C2—H2	0.9500
Cu1—N2	2.028 (3)	C3—C4	1.399 (5)
Cu1—O11	2.474 (2)	C3—H3	0.9500
Cu1—O7	2.503 (2)	C4—C5	1.374 (5)
Cu2—O13	1.933 (2)	C4—H4	0.9500
Cu2—N3	1.964 (2)	C5—C6	1.481 (5)
Cu2—N4	2.056 (3)	C6—C7	1.377 (4)
Cu2—O14	2.062 (2)	C7—C8	1.399 (5)
Cu2—O12	2.188 (2)	C7—H7	0.9500
Cu3—O13	1.951 (2)	C8—C9	1.358 (5)
Cu3—O4	1.960 (2)	C8—H8	0.9500
Cu3—N5	2.008 (3)	C9—C10	1.404 (5)
Cu3—N6	2.044 (2)	C9—H9	0.9500
Cu3—O15	2.292 (3)	C11—C12	1.404 (4)
O1—C1	1.304 (4)	C12—C13	1.376 (5)
O1—H1	0.8400	C12—H12	0.9500
O2—C10	1.324 (4)	C13—C14	1.393 (4)
O2—H2A	0.8400	C13—H13	0.9500
O3—C11	1.312 (4)	C14—C15	1.380 (4)
O4—C20	1.302 (4)	C14—H14	0.9500
O5—C21	1.321 (4)	C15—C16	1.483 (4)
O5—H5	0.8400	C16—C17	1.363 (4)
O6—C30	1.330 (4)	C17—C18	1.394 (5)
O6—H6	0.8400	C17—H17	0.9500
O7—N7	1.244 (3)	C18—C19	1.369 (4)
O8—N7	1.243 (3)	C18—H18	0.9500
O9—N7	1.277 (3)	C19—C20	1.418 (4)
O10—N8	1.255 (4)	C19—H19	0.9500
O11—N8	1.247 (4)	C21—C22	1.404 (4)
O12—N8	1.265 (4)	C22—C23	1.364 (4)
O13—H13A	0.828 (19)	C22—H22	0.9500
O14—H14A	0.823 (18)	C23—C24	1.400 (5)
O15—C31	1.452 (5)	C23—H23	0.9500
O15—H15	0.85 (4)	C24—C25	1.377 (4)
N1—C1	1.345 (4)	C24—H24	0.9500
N1—C5	1.368 (4)	C25—C26	1.472 (4)
N2—C10	1.338 (4)	C26—C27	1.379 (4)
N2—C6	1.369 (4)	C27—C28	1.385 (5)
N3—C11	1.352 (4)	C27—H27	0.9500
N3—C15	1.352 (4)	C28—C29	1.365 (5)
N4—C20	1.349 (4)	C28—H28	0.9500
N4—C16	1.373 (4)	C29—C30	1.401 (4)
N5—C21	1.337 (4)	C29—H29	0.9500
N5—C25	1.372 (4)	C31—H31A	0.9800
N6—C30	1.346 (4)	C31—H31B	0.9800
N6—C26	1.368 (4)	C31—H31C	0.9800
			
O3—Cu1—O14	92.09 (9)	C5—C4—H4	121.0
O3—Cu1—N1	169.20 (10)	C3—C4—H4	121.0
O14—Cu1—N1	94.74 (10)	N1—C5—C4	122.7 (3)
O3—Cu1—N2	92.42 (10)	N1—C5—C6	114.8 (3)
O14—Cu1—N2	171.80 (10)	C4—C5—C6	122.6 (3)
N1—Cu1—N2	81.86 (11)	N2—C6—C7	122.7 (3)
O3—Cu1—O11	81.68 (8)	N2—C6—C5	114.9 (3)
O14—Cu1—O11	81.22 (8)	C7—C6—C5	122.4 (3)
N1—Cu1—O11	91.10 (9)	C6—C7—C8	118.1 (3)
N2—Cu1—O11	106.22 (9)	C6—C7—H7	121.0
O3—Cu1—O7	83.89 (9)	C8—C7—H7	121.0
O14—Cu1—O7	86.60 (8)	C9—C8—C7	120.3 (3)
N1—Cu1—O7	104.84 (9)	C9—C8—H8	119.8
N2—Cu1—O7	87.07 (9)	C7—C8—H8	119.8
O11—Cu1—O7	160.71 (8)	C8—C9—C10	118.6 (3)
O13—Cu2—N3	177.44 (10)	C8—C9—H9	120.7
O13—Cu2—N4	98.59 (10)	C10—C9—H9	120.7
N3—Cu2—N4	81.24 (10)	O2—C10—N2	119.2 (3)
O13—Cu2—O14	90.04 (9)	O2—C10—C9	118.2 (3)
N3—Cu2—O14	88.22 (9)	N2—C10—C9	122.6 (3)
N4—Cu2—O14	132.65 (10)	O3—C11—N3	118.3 (3)
O13—Cu2—O12	91.19 (9)	O3—C11—C12	121.0 (3)
N3—Cu2—O12	91.20 (10)	N3—C11—C12	120.8 (3)
N4—Cu2—O12	114.24 (10)	C13—C12—C11	119.1 (3)
O14—Cu2—O12	112.01 (9)	C13—C12—H12	120.5
O13—Cu3—O4	91.56 (9)	C11—C12—H12	120.5
O13—Cu3—N5	93.98 (10)	C12—C13—C14	120.1 (3)
O4—Cu3—N5	169.81 (11)	C12—C13—H13	119.9
O13—Cu3—N6	168.03 (10)	C14—C13—H13	119.9
O4—Cu3—N6	91.13 (10)	C15—C14—C13	118.1 (3)
N5—Cu3—N6	81.74 (10)	C15—C14—H14	120.9
O13—Cu3—O15	98.82 (10)	C13—C14—H14	120.9
O4—Cu3—O15	94.90 (10)	N3—C15—C14	122.5 (3)
N5—Cu3—O15	92.70 (10)	N3—C15—C16	114.5 (3)
N6—Cu3—O15	92.58 (11)	C14—C15—C16	122.9 (3)
C1—O1—H1	109.5	C17—C16—N4	123.2 (3)
C10—O2—H2A	109.5	C17—C16—C15	122.3 (3)
C11—O3—Cu1	125.2 (2)	N4—C16—C15	114.5 (3)
C20—O4—Cu3	140.7 (2)	C16—C17—C18	119.2 (3)
C21—O5—H5	109.5	C16—C17—H17	120.4
C30—O6—H6	109.5	C18—C17—H17	120.4
N7—O7—Cu1	128.98 (17)	C19—C18—C17	118.7 (3)
N8—O11—Cu1	123.07 (18)	C19—C18—H18	120.6
N8—O12—Cu2	115.9 (2)	C17—C18—H18	120.6
Cu2—O13—Cu3	125.46 (12)	C18—C19—C20	120.1 (3)
Cu2—O13—H13A	101 (3)	C18—C19—H19	120.0
Cu3—O13—H13A	105 (3)	C20—C19—H19	120.0
Cu1—O14—Cu2	114.21 (11)	O4—C20—N4	121.0 (3)
Cu1—O14—H14A	109 (3)	O4—C20—C19	118.2 (3)
Cu2—O14—H14A	101 (3)	N4—C20—C19	120.9 (3)
C31—O15—Cu3	117.3 (2)	O5—C21—N5	120.4 (3)
C31—O15—H15	137 (3)	O5—C21—C22	117.6 (3)
Cu3—O15—H15	100 (3)	N5—C21—C22	122.0 (3)
C1—N1—C5	119.1 (3)	C23—C22—C21	118.9 (3)
C1—N1—Cu1	125.9 (2)	C23—C22—H22	120.6
C5—N1—Cu1	114.8 (2)	C21—C22—H22	120.6
C10—N2—C6	117.8 (3)	C22—C23—C24	119.7 (3)
C10—N2—Cu1	128.6 (2)	C22—C23—H23	120.1
C6—N2—Cu1	113.5 (2)	C24—C23—H23	120.1
C11—N3—C15	119.4 (3)	C25—C24—C23	119.0 (3)
C11—N3—Cu2	124.0 (2)	C25—C24—H24	120.5
C15—N3—Cu2	116.1 (2)	C23—C24—H24	120.5
C20—N4—C16	117.8 (3)	N5—C25—C24	121.4 (3)
C20—N4—Cu2	129.6 (2)	N5—C25—C26	115.6 (3)
C16—N4—Cu2	112.6 (2)	C24—C25—C26	123.0 (3)
C21—N5—C25	118.9 (3)	N6—C26—C27	121.2 (3)
C21—N5—Cu3	127.1 (2)	N6—C26—C25	115.3 (3)
C25—N5—Cu3	113.9 (2)	C27—C26—C25	123.5 (3)
C30—N6—C26	118.0 (3)	C26—C27—C28	119.7 (3)
C30—N6—Cu3	129.0 (2)	C26—C27—H27	120.2
C26—N6—Cu3	112.9 (2)	C28—C27—H27	120.2
O8—N7—O7	121.4 (3)	C29—C28—C27	120.2 (3)
O8—N7—O9	118.7 (3)	C29—C28—H28	119.9
O7—N7—O9	119.9 (2)	C27—C28—H28	119.9
O11—N8—O10	120.4 (3)	C28—C29—C30	117.8 (3)
O11—N8—O12	120.5 (3)	C28—C29—H29	121.1
O10—N8—O12	119.1 (3)	C30—C29—H29	121.1
O1—C1—N1	120.0 (3)	O6—C30—N6	118.7 (3)
O1—C1—C2	119.4 (3)	O6—C30—C29	118.2 (3)
N1—C1—C2	120.6 (3)	N6—C30—C29	123.1 (3)
C3—C2—C1	119.8 (3)	O15—C31—H31A	109.5
C3—C2—H2	120.1	O15—C31—H31B	109.5
C1—C2—H2	120.1	H31A—C31—H31B	109.5
C2—C3—C4	119.8 (3)	O15—C31—H31C	109.5
C2—C3—H3	120.1	H31A—C31—H31C	109.5
C4—C3—H3	120.1	H31B—C31—H31C	109.5
C5—C4—C3	118.0 (3)		
			
Cu1—O7—N7—O8	172.8 (2)	C13—C14—C15—C16	178.1 (3)
Cu1—O7—N7—O9	−6.8 (4)	C20—N4—C16—C17	2.7 (5)
Cu1—O11—N8—O10	−111.9 (3)	Cu2—N4—C16—C17	−178.7 (3)
Cu1—O11—N8—O12	68.9 (3)	C20—N4—C16—C15	−178.2 (3)
Cu2—O12—N8—O11	−9.2 (3)	Cu2—N4—C16—C15	0.4 (3)
Cu2—O12—N8—O10	171.6 (2)	N3—C15—C16—C17	171.4 (3)
C5—N1—C1—O1	−177.7 (3)	C14—C15—C16—C17	−8.2 (5)
Cu1—N1—C1—O1	7.1 (4)	N3—C15—C16—N4	−7.7 (4)
C5—N1—C1—C2	1.9 (4)	C14—C15—C16—N4	172.7 (3)
Cu1—N1—C1—C2	−173.2 (2)	N4—C16—C17—C18	−3.5 (5)
O1—C1—C2—C3	178.2 (3)	C15—C16—C17—C18	177.5 (3)
N1—C1—C2—C3	−1.5 (5)	C16—C17—C18—C19	0.9 (5)
C1—C2—C3—C4	−0.3 (5)	C17—C18—C19—C20	2.1 (5)
C2—C3—C4—C5	1.7 (5)	Cu3—O4—C20—N4	19.3 (5)
C1—N1—C5—C4	−0.5 (4)	Cu3—O4—C20—C19	−160.8 (3)
Cu1—N1—C5—C4	175.1 (3)	C16—N4—C20—O4	−179.6 (3)
C1—N1—C5—C6	179.7 (3)	Cu2—N4—C20—O4	2.1 (5)
Cu1—N1—C5—C6	−4.6 (3)	C16—N4—C20—C19	0.5 (5)
C3—C4—C5—N1	−1.3 (5)	Cu2—N4—C20—C19	−177.8 (2)
C3—C4—C5—C6	178.5 (3)	C18—C19—C20—O4	177.2 (3)
C10—N2—C6—C7	0.5 (5)	C18—C19—C20—N4	−2.9 (5)
Cu1—N2—C6—C7	176.2 (2)	C25—N5—C21—O5	179.1 (3)
C10—N2—C6—C5	−178.6 (3)	Cu3—N5—C21—O5	−3.4 (5)
Cu1—N2—C6—C5	−2.8 (3)	C25—N5—C21—C22	−0.8 (5)
N1—C5—C6—N2	4.9 (4)	Cu3—N5—C21—C22	176.7 (2)
C4—C5—C6—N2	−174.8 (3)	O5—C21—C22—C23	−178.6 (3)
N1—C5—C6—C7	−174.1 (3)	N5—C21—C22—C23	1.4 (5)
C4—C5—C6—C7	6.2 (5)	C21—C22—C23—C24	−1.1 (5)
N2—C6—C7—C8	−0.5 (5)	C22—C23—C24—C25	0.3 (5)
C5—C6—C7—C8	178.5 (3)	C21—N5—C25—C24	0.0 (5)
C6—C7—C8—C9	−0.7 (5)	Cu3—N5—C25—C24	−177.8 (3)
C7—C8—C9—C10	1.8 (5)	C21—N5—C25—C26	177.6 (3)
C6—N2—C10—O2	−178.4 (3)	Cu3—N5—C25—C26	−0.2 (4)
Cu1—N2—C10—O2	6.6 (5)	C23—C24—C25—N5	0.2 (5)
C6—N2—C10—C9	0.7 (5)	C23—C24—C25—C26	−177.2 (3)
Cu1—N2—C10—C9	−174.3 (2)	C30—N6—C26—C27	2.6 (5)
C8—C9—C10—O2	177.3 (3)	Cu3—N6—C26—C27	−173.8 (3)
C8—C9—C10—N2	−1.8 (5)	C30—N6—C26—C25	−175.0 (3)
Cu1—O3—C11—N3	67.6 (4)	Cu3—N6—C26—C25	8.6 (3)
Cu1—O3—C11—C12	−113.4 (3)	N5—C25—C26—N6	−5.7 (4)
C15—N3—C11—O3	176.9 (3)	C24—C25—C26—N6	171.9 (3)
Cu2—N3—C11—O3	−12.0 (4)	N5—C25—C26—C27	176.8 (3)
C15—N3—C11—C12	−2.0 (5)	C24—C25—C26—C27	−5.7 (5)
Cu2—N3—C11—C12	169.1 (2)	N6—C26—C27—C28	−3.2 (5)
O3—C11—C12—C13	−179.4 (3)	C25—C26—C27—C28	174.2 (3)
N3—C11—C12—C13	−0.5 (5)	C26—C27—C28—C29	1.0 (6)
C11—C12—C13—C14	2.1 (5)	C27—C28—C29—C30	1.6 (5)
C12—C13—C14—C15	−1.1 (5)	C26—N6—C30—O6	178.8 (3)
C11—N3—C15—C14	3.1 (5)	Cu3—N6—C30—O6	−5.5 (5)
Cu2—N3—C15—C14	−168.7 (3)	C26—N6—C30—C29	0.2 (5)
C11—N3—C15—C16	−176.5 (3)	Cu3—N6—C30—C29	175.9 (3)
Cu2—N3—C15—C16	11.7 (3)	C28—C29—C30—O6	179.1 (3)
C13—C14—C15—N3	−1.5 (5)	C28—C29—C30—N6	−2.2 (5)

### (II) Bis(µ3-bipyridine-2,2'-diolato-κ3O:N,N':O')tetrakis(6,6'-dihydroxybipyridine-κ2N,N')tetrakis(µ-hydroxido-κ2O:O')bis(methanol-κO)tetrakis(µ-nitrato-κ2O:O')hexacopper(II) Hydrogen-bond geometry (Å, º)

**Table:**

*D*—H···*A*	*D*—H	H···*A*	*D*···*A*	*D*—H···*A*
O1—H1···O14	0.84	1.61	2.448 (3)	175
O2—H2*A*···O3	0.84	1.66	2.499 (3)	172
O5—H5···O13	0.84	1.70	2.536 (3)	170
O6—H6···O4	0.84	1.67	2.495 (3)	168
O13—H13*A*···O9^i^	0.83 (2)	1.98 (2)	2.763 (3)	158 (4)
O14—H14*A*···O9	0.82 (2)	1.94 (2)	2.738 (3)	163 (4)
O15—H15···O10^ii^	0.85 (5)	2.42 (5)	2.790 (4)	107 (4)

Symmetry codes: (i) −*x*+1, −*y*+1, −*z*+1; (ii) −*x*+1, −*y*, −*z*+1.

## References

[bb1] Addison, A. W., Rao, T. N., Reedijk, J., van Rijn, J. & Verschoor, G. C. J. (1984). *J. Chem. Soc. Dalton Trans.* pp. 1349–1356.

[bb2] Barnett, S. M., Goldberg, K. I. & Mayer, J. M. (2012). *Nat. Chem.* **4**, 498–502. 10.1038/nchem.1350 22614386

[bb3] Blakemore, J. D., Schley, N. D., Balcells, D., Hull, J. F., Olack, G. W., Incarvito, C. D., Eisenstein, O., Brudvig, G. W. & Crabtree, R. H. (2010). *J. Am. Chem. Soc.* **132**, 16017–16029. 10.1021/ja104775j 20964386

[bb4] Bruker (2009). *APEX2*, *SAINT* and *SADABS*. Version 2009.7-0. Bruker AXS Inc., Madison, Wisconsin, USA.

[bb5] Bruker (2012). *APEX2* and *SAINT*. Version 2012.4-3. Bruker AXS Inc., Madison, Wisconsin, USA.

[bb6] DePasquale, J., Nieto, I., Reuther, L. E., Herbst-Gervasoni, C. J., Paul, J. J., Mochalin, V., Zeller, M., Thomas, C. M., Addison, A. W. & Papish, E. T. (2013). *Inorg. Chem.* **52**, 9175–9183. 10.1021/ic302448d 23387353

[bb7] Gerlach, D. L., Bhagan, S., Cruce, A. A., Burks, D. B., Nieto, I., Truong, H. T., Kelley, S. P., Herbst-Gervasoni, C. J., Jernigan, K. L., Bowman, M. K., Pan, S., Zeller, M. & Papish, E. T. (2014). *Inorg. Chem.* **53**, 12689–12698. 10.1021/ic501018a 25427106

[bb8] Groom, C. R. & Allen, F. H. (2014). *Angew. Chem. Int. Ed.* **53**, 662–671. 10.1002/anie.201306438 24382699

[bb9] Guo, H.-X., Rao, Z.-M. & Wang, Q.-H. (2007). *Acta Cryst.* E**63**, m637–m638.

[bb10] He, X. & Lu, C.-Z. (2004). *Z. Anorg. Allg. Chem.* **630**, 756–759.

[bb11] Hübschle, C. B., Sheldrick, G. M. & Dittrich, B. (2011). *J. Appl. Cryst.* **44**, 1281–1284. 10.1107/S0021889811043202 PMC3246833 22477785

[bb12] Jeffrey, G. A. (2003). *Crystallogr. Rev.* **9**, 135–176.

[bb13] Kikuchi, T. & Tanaka, K. (2014). *Eur. J. Inorg. Chem.* **2014**, 607–618.

[bb14] Luo, F., Che, Y.-X. & Zheng, J.-M. (2006). *Inorg. Chem. Commun.* **9**, 848–851.

[bb15] Macrae, C. F., Bruno, I. J., Chisholm, J. A., Edgington, P. R., McCabe, P., Pidcock, E., Rodriguez-Monge, L., Taylor, R., van de Streek, J. & Wood, P. A. (2008). *J. Appl. Cryst.* **41**, 466–470.

[bb27] Marelius, D. C., Bhagan, S., Charboneau, D. J., Schroeder, K. M., Kamdar, J. M., McGettigan, A. R., Freeman, B. J., Moore, C. E., Rheingold, A. L., Cooksy, A. L., Smith, D. K., Paul, J. J., Papish, E. T. & Grotjahn, D. B. (2014). *Eur. J. Inorg. Chem.* pp. 676–689.

[bb16] Sheldrick, G. M. (2008). *Acta Cryst.* A**64**, 112–122. 10.1107/S0108767307043930 18156677

[bb17] Sheldrick, G. M. (2009). *TWINABS.* University of Göttingen, Germany.

[bb18] Sheldrick, G. M. (2015). *Acta Cryst.* C**71**, 3–8.

[bb19] Singh, A. & Spiccia, L. (2013). *Coord. Chem. Rev.* **257**, 2607–2622.

[bb20] Sun, Y.-H., Yu, J.-H., Jin, X.-J., Song, J.-F., Xu, J.-Q. & Ye, L. (2006). *Inorg. Chem. Commun.* **9**, 1087–1090.

[bb21] Wang, C.-M., Zheng, S.-T. & Yang, G.-Y. (2009). *J. Clust Sci.* **20**, 489–501.

[bb22] Zhang, J.-P., Han, Z.-B., Chen, X.-M. & Xuebao, W. H. (2004). *Chin. J. Inorg. Chem.* **20**, 1213–1216.

[bb23] Zhang, X.-M., Tong, M.-L. & Chen, X.-M. (2002). *Angew. Chem. Int. Ed.* **41**, 1029–1031. 10.1002/1521-3773(20020315)41:6<1029::aid-anie1029>3.0.co;2-b 12491302

[bb24] Zhang, X.-M., Tong, M.-L., Gong, M.-L., Lee, H.-K., Luo, L., Li, K.-F., Tong, Y.-X. & Chen, X.-M. (2002). *Chem. Eur. J.* **8**, 3187–3194. 10.1002/1521-3765(20020715)8:14<3187::AID-CHEM3187>3.0.CO;2-9 12203348

[bb25] Zhong, Z.-G., Li, J. & Song, J.-F. (2013). *Z. Kristallogr. New Cryst. Struct* **228**, 261–262.

[bb26] Zhong, Z.-G., Feng, Y.-Q. & Zhang, P. (2013). *Acta Cryst.* C**69**, 833–836. 10.1107/S0108270113018064 23907870

